# Ligand-independent integrin **β**1 signaling supports lung adenocarcinoma development

**DOI:** 10.1172/jci.insight.154098

**Published:** 2022-08-08

**Authors:** Scott M. Haake, Erin J. Plosa, Jonathan A. Kropski, Lindsay A. Venton, Anupama Reddy, Fabian Bock, Betty T. Chang, Allen J. Luna, Kateryna Nabukhotna, Zhi-Qi Xu, Rebecca A. Prather, Sharon Lee, Harikrishna Tanjore, Vasiliy V. Polosukhin, Olga M. Viquez, Angela Jones, Wentian Luo, Matthew H. Wilson, W. Kimryn Rathmell, Pierre P. Massion, Ambra Pozzi, Timothy S. Blackwell, Roy Zent

**Affiliations:** 1 Division of Hematology/Oncology, Department of Medicine, Vanderbilt University Medical Center, Nashville, Tennessee, USA.; 2 Department of Veterans Affairs, Nashville, Tennessee, USA.; 3 Vanderbilt-Ingram Cancer Center, Nashville, Tennessee, USA.; 4 Division of Neonatology, Department of Pediatrics, Nashville, Tennessee, USA.; 5 Division of Allergy, Pulmonary, and Critical Care Medicine, Department of Medicine, Vanderbilt University Medical Center, Nashville, Tennessee, USA.; 6 Department of Cell and Developmental Biology, Vanderbilt University, Nashville, Tennessee, USA.; 7 Vindhya Data Science, Morrisville, North Carolina, USA.; 8 Division of Nephrology and Hypertension, Department of Medicine, and; 9 Vanderbilt Technologies for Advanced Genomics (VANTAGE), Vanderbilt University Medical Center, Nashville, Tennessee, USA.

**Keywords:** Oncology, Extracellular matrix, Integrins, Lung cancer

## Abstract

Integrins — the principal extracellular matrix (ECM) receptors of the cell — promote cell adhesion, migration, and proliferation, which are key events for cancer growth and metastasis. To date, most integrin-targeted cancer therapeutics have disrupted integrin-ECM interactions, which are viewed as critical for integrin functions. However, such agents have failed to improve cancer patient outcomes. We show that the highly expressed integrin β1 subunit is required for lung adenocarcinoma development in a carcinogen-induced mouse model. Likewise, human lung adenocarcinoma cell lines with integrin β1 deletion failed to form colonies in soft agar and tumors in mice. Mechanistically, we demonstrate that these effects do not require integrin β1–mediated adhesion to ECM but are dependent on integrin β1 cytoplasmic tail-mediated activation of focal adhesion kinase (FAK). These studies support a critical role for integrin β1 in lung tumorigenesis that is mediated through constitutive, ECM binding–independent signaling involving the cytoplasmic tail.

## Graphical abstract

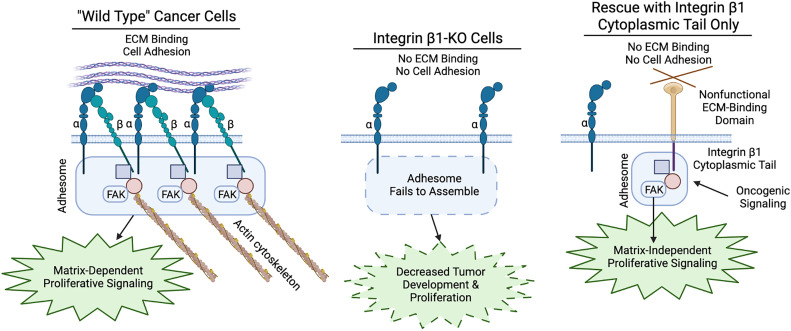

## Introduction

Integrins are the principal extracellular matrix (ECM) receptors of the cell. These transmembrane receptors form a critical mechanical link between the ECM and the cytoskeleton and, thus, facilitate cell adhesion and adhesion-dependent functions such as proliferation, migration, and invasion. There are 24 integrin heterodimers in mammals composed of 18 α and 8 β subunits (1). The extracellular domains bind distinct ECM proteins, and the cytoplasmic tails, especially those of the β subunits, bind cytoskeletal and signaling proteins. The integrin β1 cytoplasmic tail has a membrane proximal NPxY motif (where x represents any amino acid) and a membrane distal NxxY motif (2). These motifs are binding sites for multiple integrin–binding proteins and are required for integrin signaling and function (3–7) following ECM-induced integrin clustering.

Integrins are critical for cell and tissue homeostasis. Accordingly, dysregulation of integrin adhesion and signaling is associated with human disease. For example, several tumor types demonstrate increased expression of integrins relative to normal tissue (1). While much of these data are correlative, there is evidence that integrin dysregulation can directly contribute to cancer development and evolution. For example, there is increased expression of the integrin β1 subunit in lung adenocarcinoma cells relative to normal lung epithelium (8), where it promotes EGFR signaling and tumorigenesis (9). Likewise, integrin-dependent cell adhesion plays a fundamental role in the metastatic cascade (10, 11). Thus, integrins are critical contributors to the malignant phenotype.

Given their important role in cancer development and progression, integrins are recognized as potential targets for cancer treatments. Most therapeutic strategies target the interaction between integrins and the ECM, as this binding is druggable and viewed as critical for integrin functions. This strategy had some success in preclinical models (12, 13). For example, administration of an antibody targeting the extracellular domain of integrin β1 reduced cell proliferation and increased apoptosis in tumors developing from human breast cancer cell lines (14). However, similar approaches were not effective in cancer clinical trials. This was clearly demonstrated by the failure of cilengitide, a small molecule that inhibits integrin αvβ3 and αvβ5 binding to ECM, to improve outcomes of patients with glioblastoma when added to standard-of-care chemoradiation (15). Similarly, the αv integrin–targeting antibody abituzumab, combined with standard-of-care chemotherapy, failed to improve the primary endpoint of progression-free survival in a randomized phase II trial in oxaliplatin-refractory, *KRAS* WT colorectal cancer (16). Thus, despite the central role that integrins play in cancer development, integrin-targeted therapies have not been successful in improving clinical outcomes for cancer patients.

In this study, we sought to understand the role of integrin β1 in lung cancer development and to determine why inhibiting integrin-ECM interactions failed as a cancer therapeutic strategy. We demonstrate that integrin β1 promotes lung adenocarcinoma development and growth via a mechanism that is independent of integrin-ECM interactions and only requires integrin β1 cytoplasmic tail signaling. Thus, integrin β1 functions as a critical ECM-independent signaling hub in lung cancer cells, and antineoplastic drugs directed at integrins either need to target the cytoplasmic tail or proteins that bind to it.

## Results

### Deletion of integrin β1 in type 2 alveolar epithelial cells reduces tumorigenesis.

We set out to define the role of β1 integrins on lung cancer initiation and progression using the LSL–Kras-G12D mouse strain, which carries a Lox-Stop-Lox (LSL) sequence followed by the Kras-G12D point mutation allele commonly associated with human cancer. When this mouse is bred to a strain expressing Cre recombinase under control of tissue-specific promoters, the Cre recombination deletes the LSL cassette and allows expression of the mutant Kras oncogenic protein. To study the role of integrin β1 in lung cancer, we crossed these mice with integrin β1^fl/fl^ and SPC-CreER^T2^ mice. These mouse crossings were designed to simultaneously induce the LSL–Kras-G12D mutation and delete the integrin β1 subunit in type 2 alveolar (AT2) cells, the cell of origin for lung adenocarcinoma (17), in an inducible fashion. Unfortunately, tumor initiation occurred in mice never exposed to tamoxifen, suggesting constitutive activation of Cre. This precluded the use of this model from further study (Supplemental Figure 1; supplemental material available online with this article; https://doi.org/10.1172/jci.insight.154098DS1).

**Figure 1:**
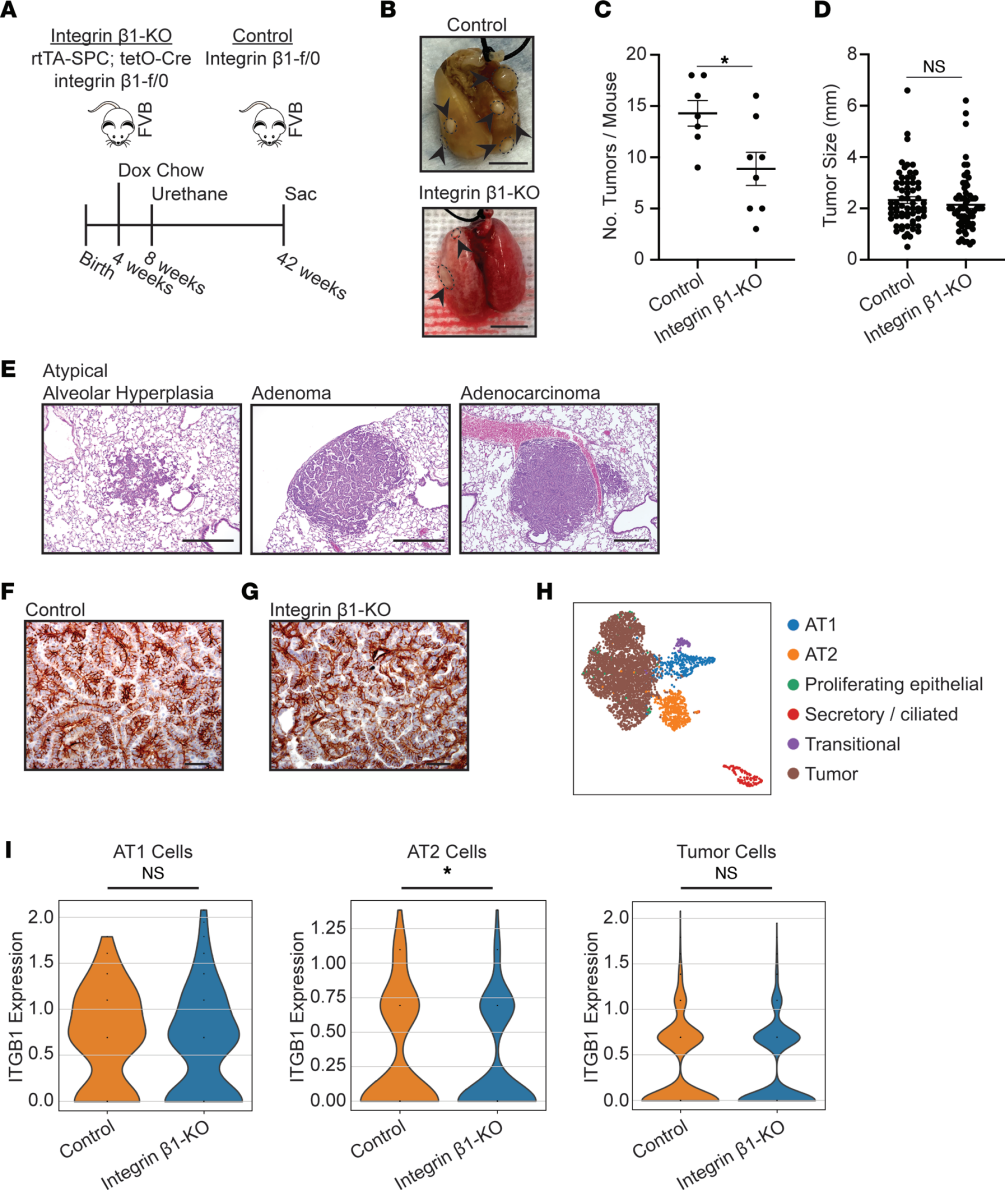
Deletion of integrin β1 in type 2 alveolar epithelial cells results in development of fewer tumors. (**A**) Tumorigenesis was initiated with urethane in integrin β1^fl/0^ mice without (control, *n* = 7) and with (integrin β1–KO, *n* = 8) dox-inducible SPC rtTA;TetO-Cre. (**B**) Representative photograph of formalin-inflated lungs (removed en bloc with heart/mediastinum) demonstrating fewer tumors in the integrin β1–KO mice relative to the control mice (arrow heads, tumor). Scale bar: 1 cm. (**C**) Quantitation of tumor count across the entire cohort. (**D**) Longest diameter of all tumors from integrin β1–KO and control mice is graphed. (**E**) Both control and integrin β1–KO mice developed lesions across the spectrum of disease, with representative photomicrographs shown of atypical alveolar hyperplasia, adenomas, and adenocarcinomas. Scale bar: 500 μm. Lesions that developed in the WT and integrin β1–KO mice were histologically indistinguishable, and the lesions shown are representative of those that developed in either strain of mouse. (**F** and **G**) FFPE tumors from control and integrin β1–KO mice were stained for integrin β1 with representative photomicrographs shown (*n* = 5). Scale bar: 50 μm. (**H**) Single-cell RNA-Seq was performed on tumors and adjacent normal tissue (tissue was pooled for *n* = 2 mice from each genotype). Uniform manifold approximation and projection (UMAP) depicting epithelial-like cells isolated from tumors or adjacent tissue from integrin β1–KO and control mouse lungs are shown. (**I**) Relative levels of integrin β1 (*Itgb1*) gene expression is shown for AT1, AT2, and tumor cells. *P < 0.05 by unpaired, 2-tailed *t* test. Data are shown as mean ± SEM.

We then made use of a urethane-induced lung cancer model in mice where the integrin β1 subunit was deleted in AT2 cells. These mice were generated by crossing integrin β1–floxed mice (β1^fl/0^) mice with a doxycycline (dox) inducible Cre recombinase under control of the surfactant protein-C promoter (SPC rtTA;TetO-Cre) (Figure 1A and Supplemental Figure 2). Although we previously showed efficient integrin β1 deletion in this model (18), we verified this again in mice fed dox chow by staining frozen sections for pro-SPC to identify AT2 cells and integrin β1. Under low magnification, AT2 cells were identified with robust, often circumferential integrin β1 staining in β1^fl/0^ (control) mice fed dox chow (Supplemental Figure 3A, left panels, white arrows). The integrin β1 staining was markedly decreased in pro-SPC^+^ cells in SPC rtTA;TetO-Cre;β1^fl/0^ (hereafter called integrin β1–KO) mice fed dox chow (Supplemental Figure 3A, right panels, white arrows). This staining was examined in more detail and quantified using 3-dimensional superresolution microscopy with reconstructions and surface plots for integrin β1 (Supplemental Figure 3B). There was more than a 2× decrease in integrin β1 staining in integrin β1–KO AT2 cells (Supplemental Figure 3B).

For tumor induction, mice were started on dox chow at 4 weeks of age, given i.p. urethane at 8 weeks, and then aged to approximately 42 weeks. Significantly fewer tumors developed in integrin β1–KO mice than β1^fl/0^ (control) mice (Figure 1, B and C). There were no size differences in the tumors (Figure 1D), and both control and integrin β1–KO mice developed lesions across the spectrum, including atypical alveolar hyperplasia, adenomas, and adenocarcinomas (Figure 1E). As the tumors in both cohorts of mice were similar in size and appearance, we investigated whether there were differences in integrin β1 expression. Although integrin β1 is significantly decreased in most AT2 cells within the normal lung of the integrin β1–KO mice (Supplemental Figure 3), integrin β1 was expressed in all tumors assessed by immunostaining (Figure 1, F and G). To verify that integrin β1 expression was similar in tumors irrespective of the genotypes, we assessed gene expression data from single-cell RNA-Seq of tumor and adjacent normal lung epithelial cells in control and integrin β1–KO mice (Supplemental Figure 4 and Figure 1H). Normal AT2 cells demonstrated a significant decrease in gene expression of *Itgb1* (integrin β1) in integrin β1–KO mice, while tumor cells from both cohorts demonstrated similar levels of integrin β1 (Figure 1I), suggesting that only AT2 cells that escaped integrin β1 deletion were able to develop into tumors. The single-cell RNA-Seq data also demonstrate robust expression of AT2 cell–specific *Sftpc* (encoding SPC) and *Sftpa1* (encoding surfactant protein A) genes in cells labeled as tumor cells (Supplemental Figure 4). We further confirmed that the tumors developed from AT2 cells as they stained positive for the AT2 cell marker pro-SPC (Supplemental Figure 5). Taken together, these data suggest that integrin β1 expression is required for tumorigenesis in this carcinogen-induced lung cancer model.

### Human lung adenocarcinoma cells require expression of integrin β1 to form colonies and tumors.

**Figure 2:**
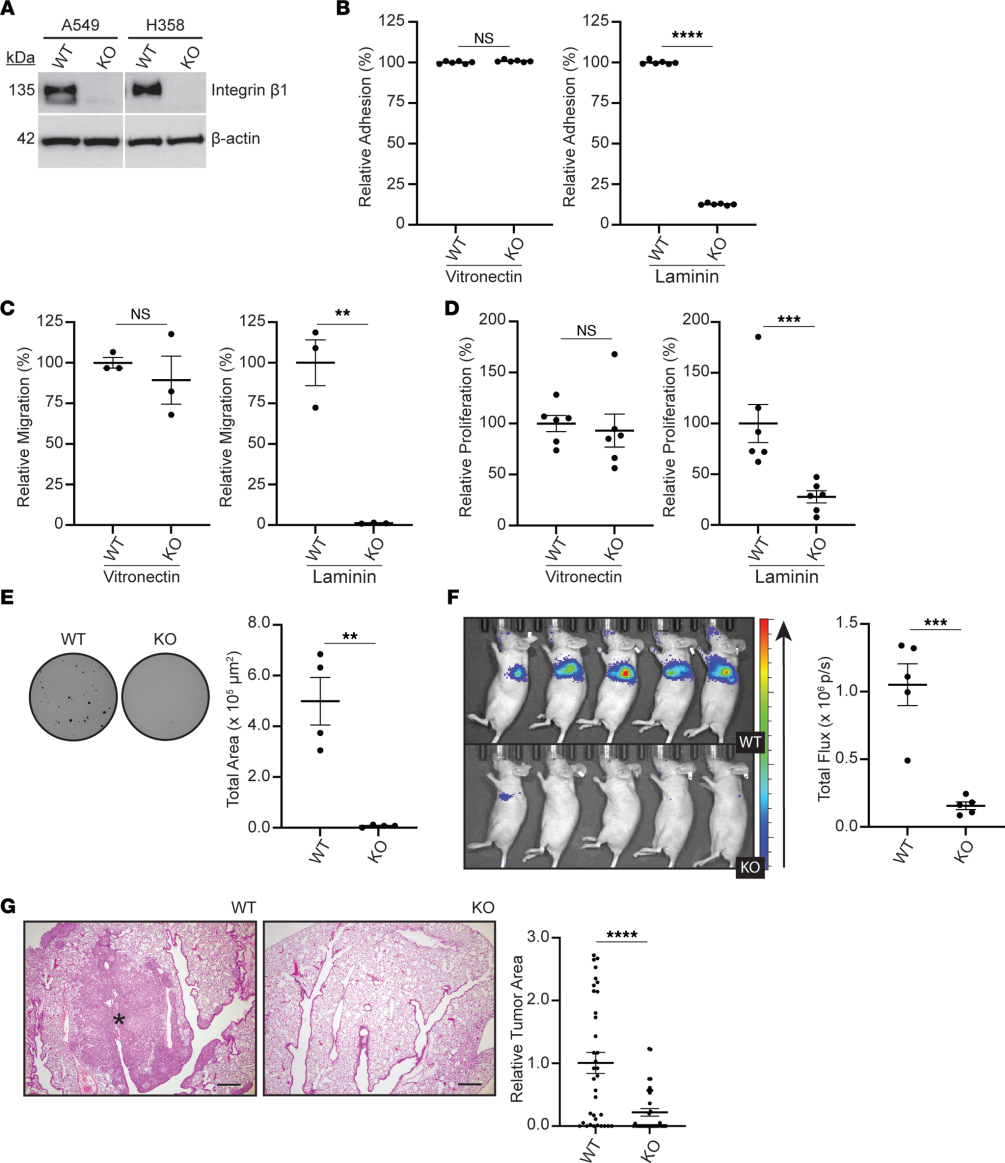
Deletion of integrin β1 in A549 human lung cancer cells results in decreased colony formation and tumor development. Integrin β1 is deleted in A549 and H358 human lung adenocarcinoma cells using CRISPR/Cas9 (KO). (**A**) Lysates from WT and KO were analyzed by Western blot for levels of integrin β1. The solid white lines represent lane splicing from the same gel. The WT and KO A549 cells were plated on integrin β1–independent (vitronectin) and integrin β1–dependent (laminin I) matrices. (**B**–**D**) Relative adhesion, migration, and proliferation measured as BrdU-incorporation is graphed for WT and integrin β1–KO cells (*n* = 3 replicates). (**E**) The WT and integrin β1–KO A549 cells were plated in soft agar. Representative photomicrographs of the wells and colony surface area quantification are shown (*n* = 3 replicates, each replicate consisting of 6 wells, representative data from one replicate shown). Luciferase-tagged A549 WT and integrin β1–KO cells were injected into the left lung of athymic mice. (**F** and **G**) After 45 days, tumor burden was quantified via luciferin injection and measurement of bioluminescence (**F**) and relative surface area as measured by microscopy (**G**) (representative photomicrographs shown, asterisk denotes tumor). Scale bar: 20 μm. *n* = 5 mice for each genotype, left lung from each mouse was sectioned every 100 μm times 7 sections. **P < 0.01; ***P < 0.001; ****P < 0.0001 by unpaired, 2-tailed *t* test. Data are shown as mean ± SEM

Since the urethane model suggested integrin β1 is required for lung tumor initiation, this was investigated further in lung adenocarcinoma cell lines, where integrin β1 was genetically downregulated. We utilized the *KRAS-*mutated human lung adenocarcinoma cell lines A549 and H358 and deleted *ITGB1* (integrin β1) using CRISPR/Cas9. The results obtained were similar in both cell lines; thus, we show data for the A549 cells in the main figures (Figure 2) and H358 cells in the supplement (Supplemental Figure 6). Deletion of integrin β1 was confirmed by Western blot (Figure 2A). Classical integrin β1–dependent functions such as adhesion, migration, and proliferation were maintained in the WT but not integrin β1–KO A549 cells on the integrin β1–dependent matrix laminin I (Figure 2, B–D). WT and integrin β1–KO A549 cells behaved similarly when they were plated on the integrin β1–independent matrix vitronectin (Figure 2, B–D). Surprisingly, the integrin β1–KO A549 cells also demonstrated decreased colony formation in the adhesion-independent soft agar assay, suggesting that nonadherent cancer cells required integrin-dependent signaling to form colonies (Figure 2E). Next, we injected the A549 cells into the lung parenchyma of athymic mice. The integrin β1–KO A549 cells demonstrated decreased tumor formation in the lungs when evaluated by bioluminescence (Figure 2F) and histology (Figure 2G). These data indicate that integrin β1 signaling is required for tumor development in an orthotopic model of lung cancer.

### Integrin β1 regulates gene expression in a matrix-dependent and -independent manner.

**Figure 3:**
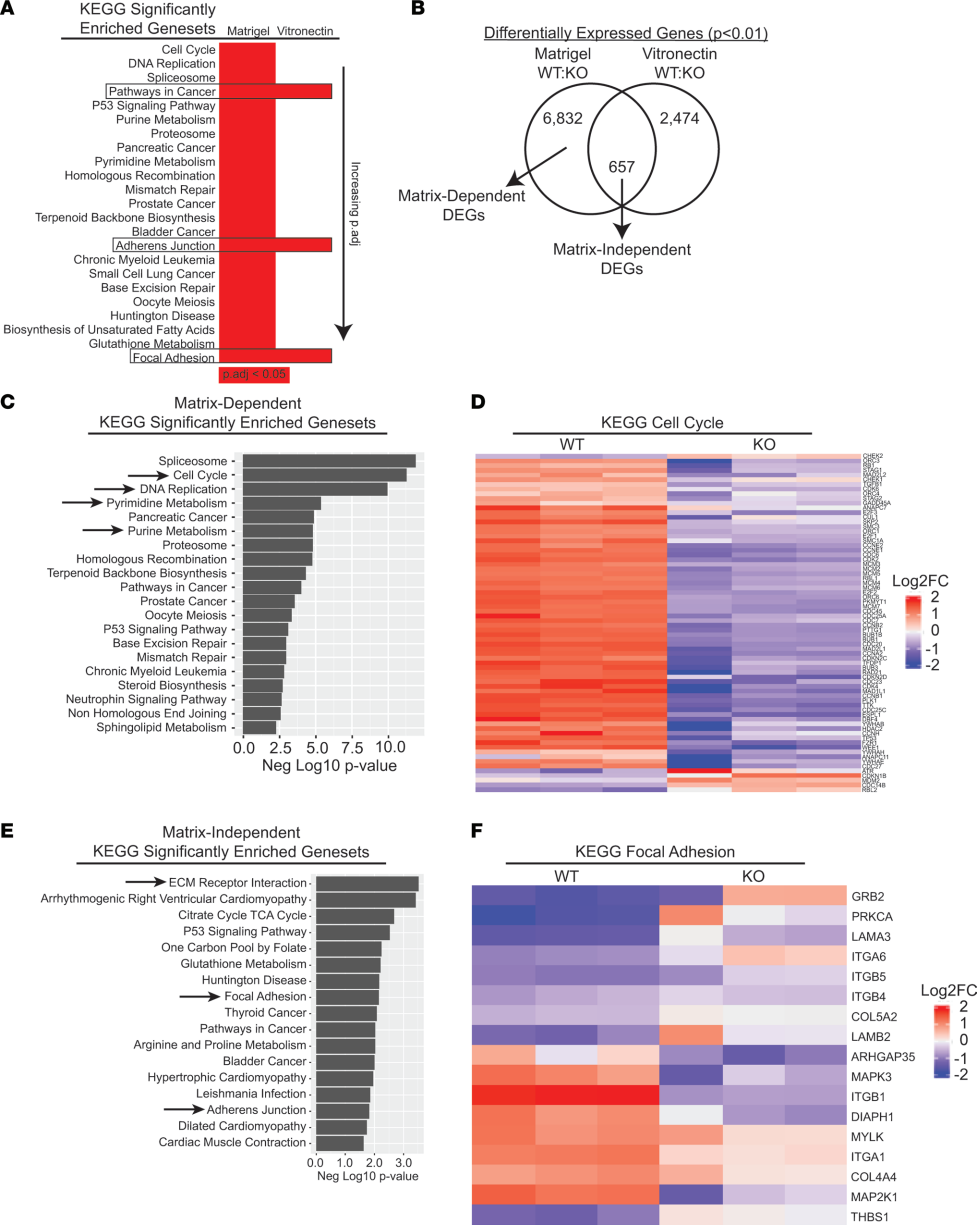
Differential gene expression in WT and integrin β1–KO A549 cells. (**A**) WT and integrin β1–KO A549 cells were plated on the integrin β1–dependent matrix Matrigel or the integrin αv-dependent matrix vitronectin and gene expression measured by RNA-Seq (*n* = 3 replicates for each cell line). Gene set enrichment analysis was performed using differentially expressed genes (DEGs, *P* < 0.01). Shown are the significant pathways (*P*_adj_ < 0.05) from cells plated on Matrigel and corresponding *P*_adj_ values for cells plated on vitronectin (red square, *P*_adj_ < 0.05; white square, not significant). (**B**) Venn diagram demonstrates DEGs exclusive to cells plated on Matrigel that normalize in cells plated on vitronectin (matrix-dependent DEGs), as well as DEGs shared by cells plated on either matrix whose directionality aligns (e.g., increased in integrin β1–KO cells relative to WT cells on both matrices; matrix-independent DEGs). (**C**) Gene set enrichment analysis was performed on matrix-dependent DEGs, demonstrating several significant gene sets including proliferation-associated gene sets (arrows). (**D**) Representative heatmap of significant gene set from the matrix-dependent genes (KEGG_CELL_CYCLE). (**E**) Gene set enrichment analysis was also performed matrix-independent DEGs, demonstrating several significant gene sets including cell adhesion and ECM-related genes (arrows). (**F**) Representative heatmap of significant gene set from the matrix-independent genes (KEGG_FOCAL_ADHESION).

To understand why integrin β1 is necessary for tumor development, we performed RNA-Seq on WT and integrin β1–KO A549 cells. Cells were plated on either Matrigel that allows integrin-dependent cell adhesion in WT integrin β1^+^ cells but not integrin β1–KO cells, or vitronectin that allows integrin αv–dependent adhesion of both WT and integrin β1–KO cells. The gene expression of WT and integrin β1–KO cells were compared, and differentially expressed genes (DEGs) were identified (*P* < 0.01). Kyoto Encyclopedia of Genes and Genomes (KEGG) pathway analysis was performed on these DEGs from cells plated on Matrigel or vitronectin and ranked (top, most significant; bottom, less significant; Figure 3A). Many of the significantly different gene sets found in integrin β1–KO cells plated on Matrigel were no longer different when the cells were plated on vitronectin, suggesting that adhesion via αv integrins is sufficient to normalize these gene expression changes (Figure 3A). There were also DEG sets in cells plated on either Matrigel or vitronectin, suggesting that expression of these genes requires integrin β1 expression but not integrin β1–dependent ECM interactions (see black boxes in Figure 3A). To explore this biology further, we identified DEGs shared by cells plated on Matrigel and vitronectin (657 matrix-independent DEGs) and DEGs exclusive to cells plated on Matrigel (6832 matrix-dependent DEGs) (Figure 3B). Pathways associated with cell proliferation, including KEGG_CELL_CYCLE, KEGG_DNA_REPLICATION, KEGG_PYRIMIDINE_METABOLISM, and KEGG_PURINE_METABOLISM (arrows; Figure 3C) predominated in the matrix-dependent DEGs, and a heatmap for KEGG_CELL_CYCLE demonstrated that gene expression was decreased in the integrin β1–KO cells (Figure 3D). When we examined the pathways enriched in a matrix-independent manner, the major DEGs included those pertaining to the ECM and cell adhesion (KEGG_ECM_RECEPTOR_INTERACTION, KEGG_FOCAL_ADHESION, KEGG_ADHERENS_JUNCTION, arrows; Figure 3E), and the heatmap for KEGG_FOCAL_ADHESION demonstrated robust changes in several ECM-associated genes in the integrin β1–KO cells (Figure 3F). These data implicate integrin β1 in the regulation of cancer-relevant genes via mechanisms that are both dependent and independent of integrin-mediated cell adhesion to ECM.

### Integrin β1 regulates growth factor–dependent signaling required for colony formation.

**Figure 4:**
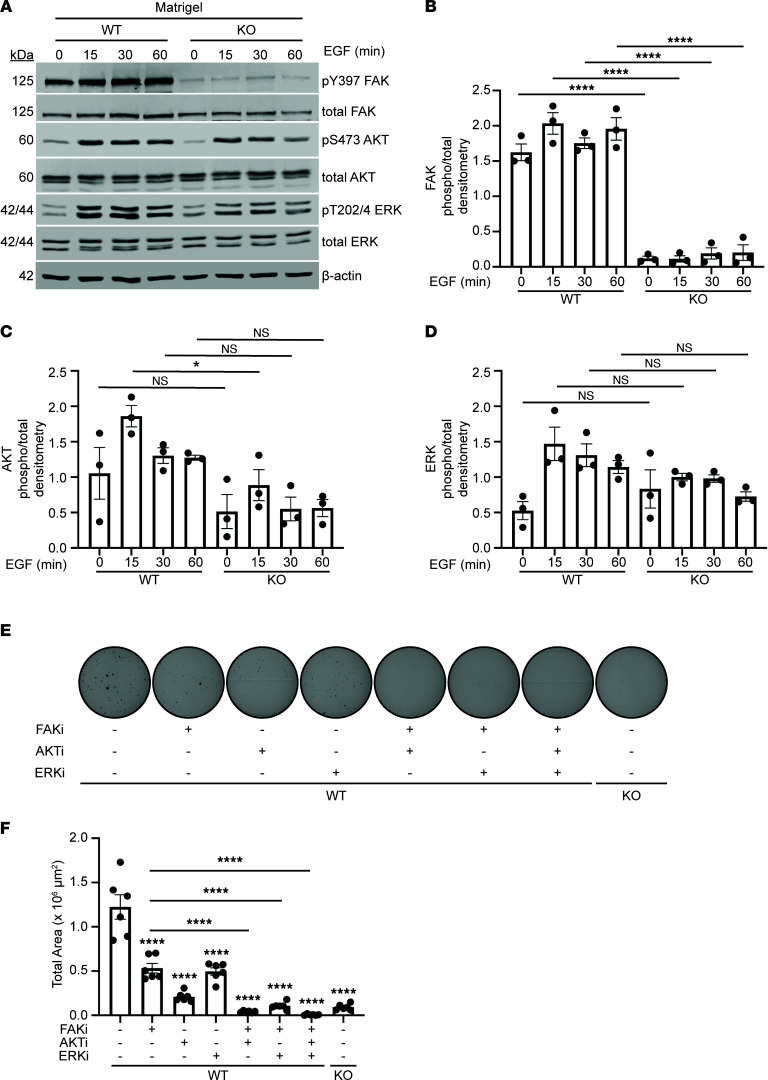
Deletion of integrin β1 results in decreased growth factor–dependent signaling. (**A**) A549 cells were plated on the integrin β1–dependent matrix Matrigel and stimulated with EGF (1 ng/mL) for 0, 15, 30, or 60 minutes, and then lysates were analyzed by Western blot for levels of total and activated FAK, AKT, and ERK. (**B**–**D**) Results were quantified via densitometry (average of *n* = 3 replicates). WT A549 cells were treated with inhibitors of FAK (defactinib, 1.0 μM), AKT (MK-2206, 0.1 μM), and ERK (SCH772984, 0.01 μM) alone or in combinations. Integrin β1–KO cells included as negative control. (**E** and **F**) Representative photomicrographs and surface area quantification of colonies are shown (*n* = 3 replicates, each replicate consisting of 6 wells, representative data from one replicate shown). All comparisons include DMSO-treated cells (i.e., those not treated with FAKi, AKTi, or ERKi) unless comparison otherwise marked by bar. *P < 0.05; ****P < 0.0001 by Sidak’s multiple-comparison test. Data are shown as mean ± SEM.

We next tested whether integrin β1 regulates cancer cell proliferation signaling pathways, as suggested by the gene expression data. We utilized EGF, as it is a well-known growth factor that drives lung tumorigenesis and because activating mutations in the EGF receptor are driver mutations in some lung tumors (19, 20). We treated WT and integrin β1–KO A549 cells plated on Matrigel with EGF and then measured activation of key cell proliferation signaling molecules, AKT and ERK, as well as FAK, a known downstream target of integrin β1 that is stimulated in adherent proliferating cells. Interestingly, there was decreased phosphorylation of FAK and AKT in integrin β1–KO A549 cells prior to treatment with EGF, suggesting that integrin β1 signaling plays a role in the basal activation of these pathways in *KRAS-*mutated lung cancer cells. We further noted that EGF treatment of WT A549 cells resulted in increased FAK, ERK, and AKT phosphorylation (Figure 4, A–D), which was less robust in the integrin β1–KO A549 cells. As the difference in basal and EGF-induced FAK activation between the cell lines was highly significant, we suspected this was a major mechanism whereby integrin β1 regulates tumor cell growth and proliferation. To test this hypothesis, we treated WT and integrin β1–KO A549 cells plated in soft agar with the FAK tyrosine kinase inhibitor (TKI) defactinib, as well as inhibitors to AKT and ERK (cell signaling proteins that commonly transmit important mitogen signaling in cells). First, drug doses were selected that result in a robust, significant decrease in colony formation (Supplemental Figure 7). Next, we confirmed that these inhibitors reduced phosphorylation of the target kinases at the specified doses in WT A549 cells (Supplemental Figure 8). Finally, WT A549 cells were treated with single inhibitor or combinations (Figure 4, E and F). FAK inhibition failed to completely inhibit colony formation, and the addition of either the AKT or ERK inhibitor reduced colony formation further. These data suggest that FAK provides oncogenic signaling independently of AKT and ERK.

### The integrin β1 cytoplasmic tail is necessary and sufficient for tumor formation.

**Figure 5:**
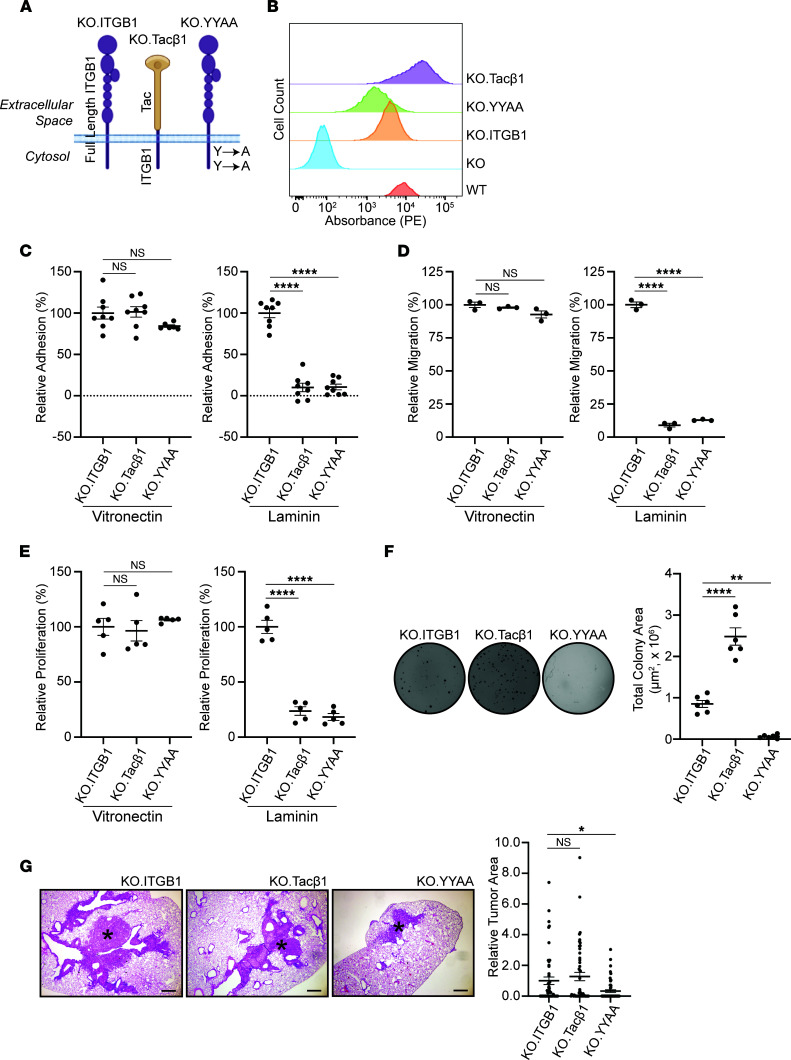
Expression of the integrin β1 cytoplasmic tail in cells lacking endogenous integrin β1 restores colony and tumor formation. (**A**) Full-length integrin β1 (ITGB1), the cytoplasmic domain of integrin β1 fused to the extracellular domain of Tac (Tacβ1), and full-length integrin β1 with Y783A and Y795A mutations (YYAA) were reexpressed in integrin β1–KO A549 cells. Panel made with assistance from www.biorender.com (**B**) Surface expression of transfected proteins was measured via flow cytometry by targeting the extracellular domain of integrin β1 (KO.ITGB1, KO.YYAA) or Tac (KO.Tacβ1). (**C**–**E**) Cells were evaluated for adhesion, migration, and proliferation on vitronectin and laminin I (*n* = 3 replicates). (**F**) A soft agar colony formation assay was performed using the KO.ITGB1, KO.Tacβ1, and KO.YYAA cells. Representative photomicrographs are shown, and data are quantified (*n* = 3 replicates, each replicate consisting of 6 wells, representative data from 1 replicate shown). (**G**) Cells were injected into the left lung of athymic mice (KO.ITGB1, *n* = 10 mice; KO.Tacβ1, *n* = 10 mice; KO.YYAA, *n* = 11 mice). Mice were sacrificed, and histologic evaluation was performed to determine whether cells formed tumors (representative photomicrographs shown, asterisk denotes tumor). Scale bar: 20 μm. Left lung from each mouse was sectioned every 100 μm times 5 sections. *P < 0.05; **P < 0.01; ****P < 0.0001 by Sidak’s multiple-comparison test. Data are shown as mean ± SEM.

Since integrin β1 is required for colony formation in soft agar, it likely mediates its effects by an adhesion-independent mechanism. We therefore generated integrin β1–KO A549 cells where we introduced either the full-length integrin β1 subunit (KO.ITGB1), a chimeric protein consisting of an integrin β1 cytoplasmic tail fused to the extracellular, and transmembrane domains of the IL-2 receptor (KO.Tacβ1) (5) or an integrin β1 subunit with Y-to-A cytoplasmic tail mutations at residues Y783 and Y795 that disrupt integrin signaling (KO.YYAA) (Figure 5A) (4, 5, 21, 22). These cells were flow sorted to achieve cell populations with comparable surface expression of these proteins (Figure 5B). As expected, the KO.ITGB1, KO.Tacβ1, and the KO.YYAA cells demonstrated similar adhesion, migration, and proliferation on the integrin β1–independent matrix vitronectin (Figure 5, C–E). By contrast, the KO.Tacβ1 and KO.YYAA cells demonstrated decreased adhesion, migration, and proliferation relative to the KO.ITGB1 cells when plated on the integrin β1–dependent matrix laminin I (Figure 5, C–E). Despite the inability to bind ECM, the KO.Tacβ1 cells formed robust colonies in soft agar and tumors in mice. The KO.YYAA cells formed almost no colonies in soft agar and significantly less tumor burden in mice (Figure 5, F and G). These data support the conclusion that a functional integrin β1 cytoplasmic tail promotes colony and tumor formation, irrespective of cell adhesion.

### The integrin β1 cytoplasmic tail is sufficient for proliferative gene expression signatures and FAK activation.

**Figure 6:**
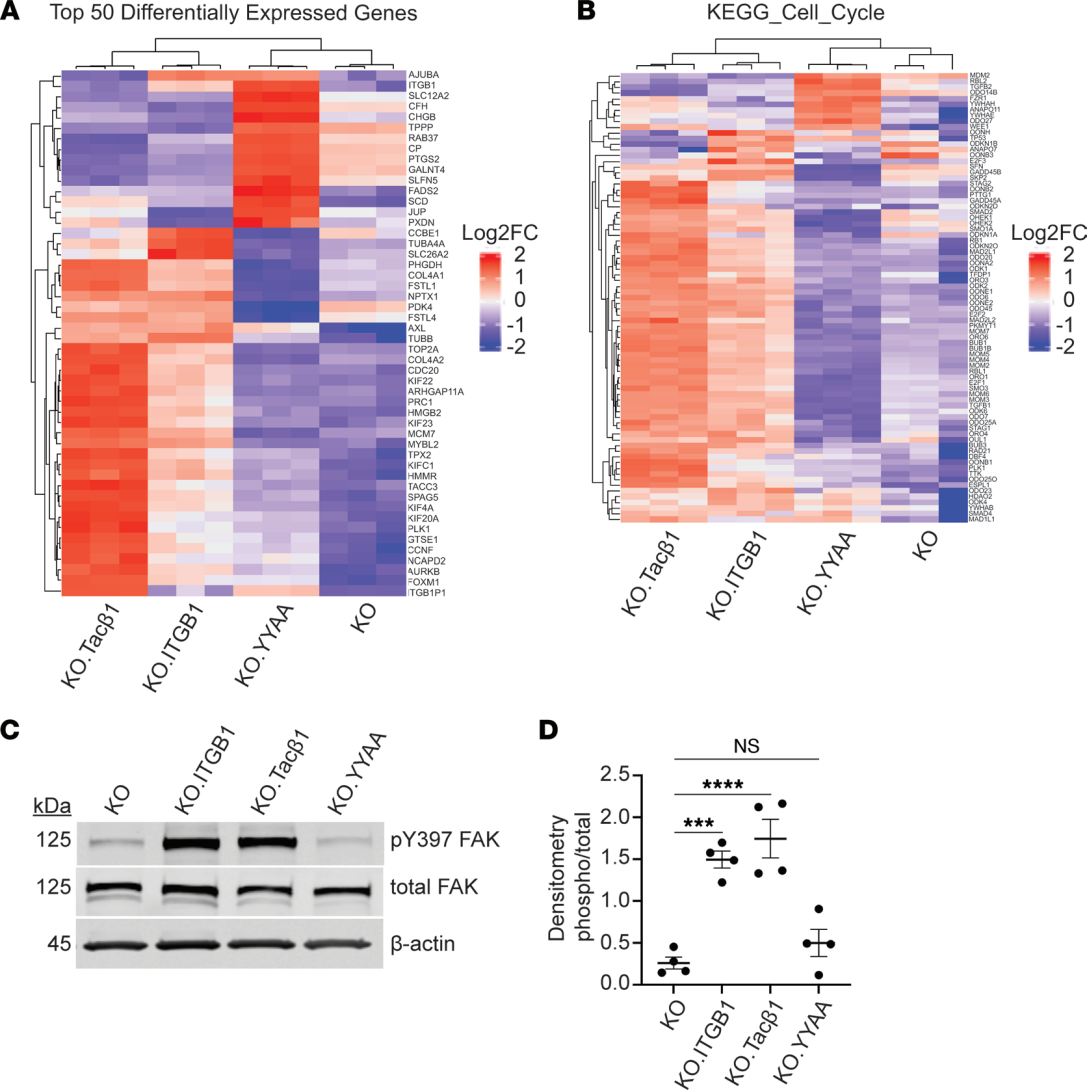
Integrin β1 cytoplasmic tail expression promotes proliferative gene expression signatures and FAK activation. (**A**) Transcriptomic gene expression was evaluated in KO, KO.ITGB1, KO.Tacβ1, and KO.YYAA cells, and unsupervised clustering was performed (*n* = 3 replicates for each cell line). (**B**) Expression of genes in the KEGG_CELL_CYCLE gene signature are increased in the KO.ITGB1 and KO.Tacβ1 cell lines relative to the KO and KO.YYAA cell lines. (**C** and **D**) Cell lysates were analyzed by Western blot for levels of total and pY397 FAK and quantified by densitometry (*n* = 4 replicates). ***P < 0.001; ****P < 0.0001 by Sidak’s multiple-comparison test. Data are shown as mean ± SEM.

We next assessed whether the integrin β1 tail was sufficient to reconstitute the gene expression profile of cells with full-length integrin β1 when plated on the integrin β1–dependent matrix Matrigel. A549 cells with a functional integrin β1 cytoplasmic tail (KO.ITGB1, KO.Tacβ1) demonstrated similar expression patterns for the top 50 DEGs (Figure 6A). These cells demonstrated higher expression of genes from the KEGG_CELL_CYCLE gene set than the cell lines lacking a functional integrin β1 cytoplasmic tail (integrin β1–KO, KO.YYAA; Figure 6B). They also demonstrated higher levels of FAK phosphorylation than the KO.YYAA cells and integrin β1–KO cells (Figure 6, C and D). These data suggest that a functional integrin β1 cytoplasmic tail is sufficient to restore expression of matrix-dependent cell cycle–related genes and activate FAK in integrin β1–KO lung adenocarcinoma cells lacking integrin-mediated adhesion to ECM.

### Integrin β1 expression in human lung tumors correlates with tumor size, survival, and cancer-associated gene signatures.

**Figure 7:**
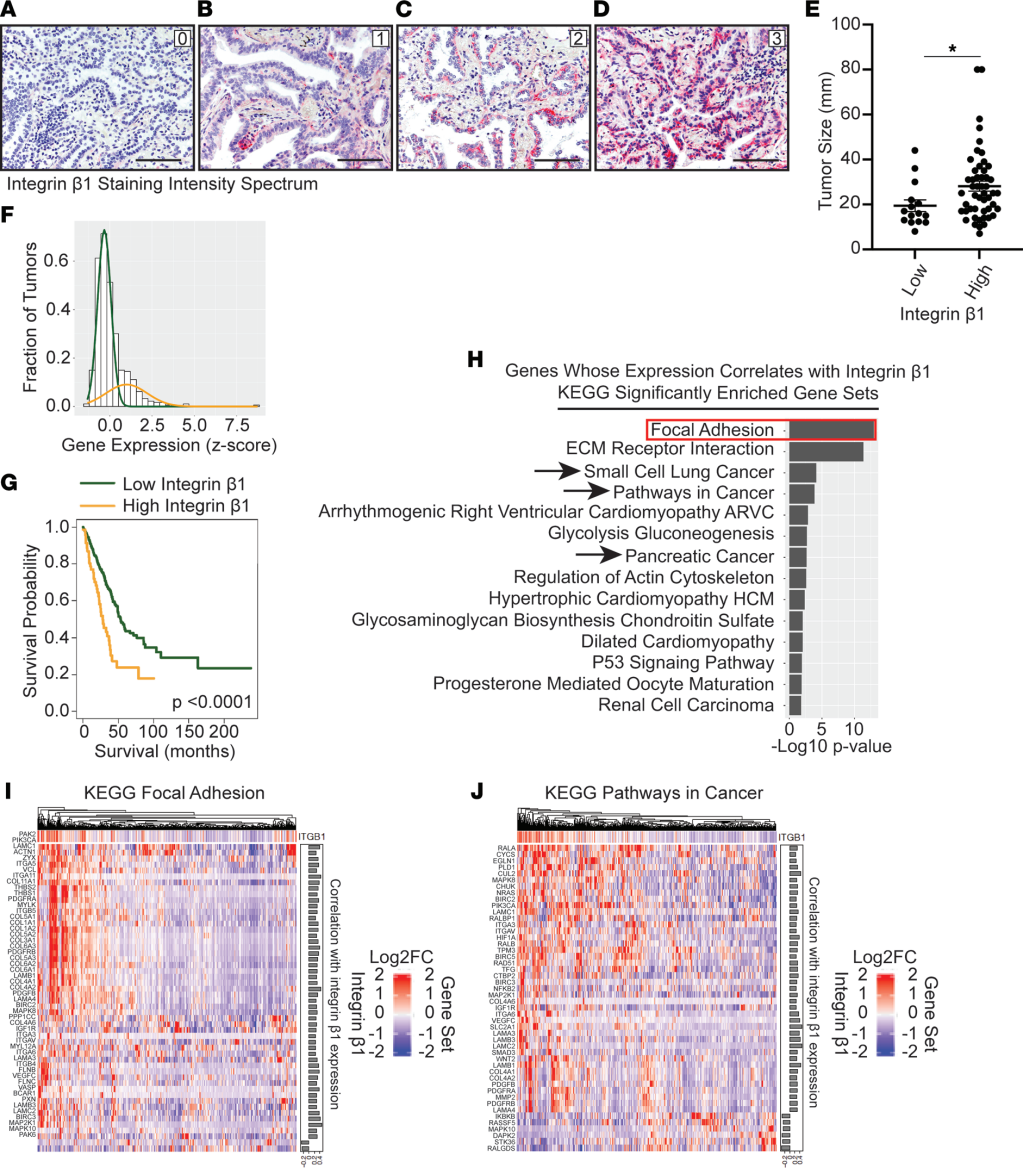
Increased integrin β1 protein and gene expression correlates with increased tumor size, poor survival, and increased expression of cancer-associated gene sets in human lung adenocarcinoma. (**A**–**D**) Tissue microarray including 65 human lung adenocarcinomas was stained for integrin β1 (red), and expression was quantified by a pathologist (labeled 0–3). (**E**) The size of tumors with high (scores 2–3 staining intensity) versus low integrin β1 protein expression (scores 0 and 1 staining intensity) was compared (mean is graphed for each group). (**F**) Gaussian mixture modeling was performed and identified tumor groups with increased (orange) and decreased (green) *ITGB1* (integrin β1) gene expression. (**G**) Overall survival was evaluated via Kaplan-Meier curve analysis in the integrin β1–high and –low groups. (**H**–**J**) Genes were identified whose expression correlates with integrin β1 expression; Pathway enrichment analysis of integrin β1–correlated genes demonstrates strong correlation with several gene expression signatures, including focal adhesion (**H** [red box] and **I**) and cancer-associated pathways (**H** [black arrows] and **J**).

The data gathered from our mouse and human cell models of lung adenocarcinoma suggest that integrin β1 is important for tumor development. We therefore assessed its relevance to human health by determining whether a similar correlation is observed in human tumors. IHC for integrin β1 was performed on a tissue microarray (TMA) consisting of 65 clinically annotated human lung adenocarcinomas. Tumor and patient characteristics are summarized (Supplemental Table 1). The stained TMA was reviewed by a pathologist, and staining intensity was scored on a scale of 0–3 (Figure 7, A–D). Integrin β1 was expressed in all molecular subtypes of lung adenocarcinoma, though expression was lower in *EGFR-*mutated tumors (*n* = 17, mean 1.4 ± SD 0.7) relative to *KRAS-*mutated tumors (*n* = 40, mean 1.9 ± SD 0.8, *P* = 0.03) and all other tumors (*n* = 8, mean 2.1 ± SD 0.5, *P* = 0.04) (data not shown). Like previous studies where integrin β1 expression correlates with recurrence-free survival (RFS) and overall survival (OS) in lung adenocarcinoma (8), our study demonstrates a trend toward improved RFS and OS in patients with low (tumors scored as 0–1 staining intensity) integrin β1–expressing tumors (Supplemental Figure 9). In addition, tumors with relatively higher (tumors scored as 2–3 staining intensity) integrin β1 expression were larger than those with lower integrin β1 expression (Figure 7E). Thus, integrin β1 expression correlates with large tumors and worse outcomes in patients.

We next performed Gaussian mixture modelling on the lung adenocarcinoma TCGA RNA-Seq data to evaluate gene expression patterns in human tumors (19). Both *ITGB1*-high (integrin β1*–*high) and integrin β1*–*low populations of tumors were identified (Figure 7F), and the integrin β1–high tumors exhibit decreased survival (consistent with other similar studies; ref. 23) (Figure 7G). Genes whose expression correlated with integrin β1 (Spearman’s correlation > 0, *q* value < 0.001) were identified. When we performed KEGG analysis, pathways that reflect classical integrin adhesion–dependent biology such as KEGG_FOCAL_ADHESION (Figure 7, H and I) were enriched with integrin β1*–*correlated genes. In addition, gene sets associated with aggressive and highly proliferative cancers that were enriched in the integrin β1–expressing cancer cell lines, including KEGG_SMALL_CELL_LUNG_CANCER, KEGG_PATHWAYS_IN_CANCER, and KEGG_PANCREATIC_CANCER, were correlated with integrin β1 expression in the human tumors (Figure 3 and Figure 7, H and J). Consistent with our findings in mouse and cell line models, these studies suggest that integrin β1 promotes tumor growth in human lung adenocarcinoma.

## Discussion

The mechanism whereby integrins promote aggressive tumor biology has classically focused on integrin-ECM binding, which facilitates cell adhesion and proliferative signaling. However, integrin-targeted therapeutics that inhibit integrin-ECM binding have failed to improve clinical outcomes in cancer patients. In the current study, we deleted the integrin β1 subunit (resulting in no expression of integrin β1–containing heterodimers) in both chemical carcinogen and cell line–based models of lung adenocarcinoma. We demonstrate that integrin β1 signaling is necessary for tumor development in mice. Next, we showed that the integrin β1 cytoplasmic tail is sufficient for integrin β1–dependent FAK activation, gene expression, and tumor development. Thus, we conclude that integrin β1 is a signaling hub for lung tumor development and proliferation that utilizes its cytoplasmic tail by mechanisms that do not require integrin-ECM binding. These data suggest that future strategies to inhibit integrins in cancer should target cytoplasmic tail–dependent signaling.

When we tested the role of integrin β1 during lung tumorigenesis by developing an autochthonous mouse model using the Cre-Lox system and the carcinogen urethane, we found that the few tumors that developed in the integrin β1–KO mice invariably expressed integrin β1. The most likely explanation for this observation is that the AT2 cells with incomplete deletion of integrin β1 exposed to urethane undergo clonal expansion and develop into lung adenocarcinomas. This results in fewer integrin β1^+^ tumors in integrin β1–KO mouse that are a similar size to those that develop in control mice. A similar phenomenon of “breakthrough” carcinogenesis in integrin β1–KO mouse has been described in a breast cancer model, resulting in integrin β1^+^ tumors developing in integrin β1–KO mice (24). Another explanation for the formation of tumors in integrin β1–KO mice is that the tumors develop from SPC^–^ cells; however, our data show that the tumor cells express SPC protein, making this possibility unlikely. Another possible but even more unlikely explanation for the formation of tumors in integrin β1–KO mice is that integrin β1 is dispensable for tumor formation. In this case, we would have expected to observe some integrin β1–null tumors; however, every tumor that developed in mice expressed integrin β1. As “Cre-escape” was a limitation of this model, we utilized a xenograft model with complete integrin β1 deletion in tumor cells to demonstrate the requirement of β1 integrin in lung tumor development.

Our findings that integrin β1 is required for tumor formation in both chemical-induced and human cell line lung adenocarcinoma models are consistent with other studies demonstrating that integrin β1 promotes tumor formation. For example, KrasLA2 mice, which carry an oncogenic mutation in *Kras* that spontaneously activates and leads to lung tumor formation, were crossed with integrin α1–null mice, resulting in deletion of integrin α1β1 (25). The integrin α1–null mice demonstrated improved survival, and the integrin α1–null tumor cells demonstrated decreased cell adhesion, ERK-activation, and tumorigenicity relative to controls due to decreased classical integrin-mediated signaling upon collagen binding. There was also decreased tumor formation in a polyomavirus middle T–driven (PyMT-driven) breast cancer model with inducible KO of integrin β1 in breast epithelium (24). Integrin β1 was also necessary for tumor development and growth in a mouse model of pancreatic neuroendocrine tumor (26). The only mouse model suggesting integrin β1 can function as a tumor suppressor is the transgenic adenocarcinoma of mouse prostate (TRAMP) adenocarcinoma mouse model (27), where tumorigenesis is driven by prostate-specific expression of SV40 early T/t antigen genes. Deletion of integrin β1 in the prostate epithelium resulted in an increased percentage of prostate gland involved by tumor and increased tumor cell proliferation, though the mechanisms are not known. Our results with the integrin β1–null A549 and H358 cells were more dramatic than those seen in our chemical-induced lung adenocarcinoma model, likely due to the complete integrin β1 deletion achieved in the cell lines. While our data are consistent with the role of integrin β1 in mediating cancer cell line invasion, migration, and metastasis on ECM (1, 28, 29), the inability of the integrin β1–KO cells to form colonies in soft agar suggests an adhesion-independent mechanism, as well.

The integrin β1–KO lung cancer cells exhibited decreased FAK, AKT, and ERK phosphorylation when compared with control cells, and this difference was most prominent for FAK. Pharmacologic inhibition of FAK also inhibited colony formation of WT cancer cells in soft agar. FAK is a nonreceptor protein tyrosine kinase downstream of integrins that regulates cell signaling and gene transcription, which in turn controls cell adhesion, migration, proliferation, and survival (30). FAK regulates gene expression via its kinase-dependent function in focal adhesion complexes localized to the plasma membrane or endosomal complexes (31), and it also translocates to the nucleus where it regulates gene expression independently of its kinase activity (30). FAK is frequently overexpressed in tumors and promotes several important malignant features, including cancer stemness, epithelial-to-mesenchymal transition, and resistance to anticancer therapies (30). Increased phosphorylated FAK is observed in both non-small cell and small cell lung cancer relative to normal lung (32). In mice with mutant *Kras* and deletion of *Cdkn2a* in lung epithelial cells, lung tumors develop with activation of ERK, RHOA, and FAK, and subsequent deletion or pharmacologic inhibition of FAK resulted in tumor regression (33). Treatment of A549 cells in this study with FAK inhibitors in combination with either AKT or ERK inhibitors resulted in decreased soft agar colony formation relative to treatment with a single inhibitor, suggesting that FAK provides oncogenic signaling that may be independent of AKT and ERK. There are several candidate pathways that could be contributing to FAK-dependent colony formation via AKT/ERK-independent mechanisms. For example, pharmacologic inhibitors of FAK have been shown to promote its translocation to the nucleus (34), where FAK promotes ubiquitylation and degradation of p53 and restriction of p53 tumor-suppressive functions (35–37). Thus, our study is consistent with prior work that identifies FAK activation as a key component that promotes lung adenocarcinoma development. While there may be many scenarios whereby FAK is activated in cancers, our models suggest that the integrin β1 cytoplasmic tail is necessary for FAK activation.

The integrin β1 cytoplasmic tail restored tumor formation, signaling (including FAK phosphorylation), and gene expression patterns to those seen in integrin β1^+^ cells. The integrin β1 cytoplasmic tail was previously shown to be sufficient to reconstitute cell functions like paracellular transport in integrin β1–null kidney epithelial proximal tubule cells (5). Other studies with Tacβ1 have been performed in the cells with retained endogenous integrin β1 expression, and their results have been inconsistent. For example, mouse fibroblast cells engineered to express Tacβ1 exhibit constitutive, adhesion-independent FAK activation (consistent with our results) (38), whereas in CHO cells the Tacβ1 chimeric protein inhibited cell spreading and decreased SRC and FAK phosphorylation due to sequestration of integrin β1 cytoplasmic tail binding proteins (39). In addition, others demonstrated that FAK tethered to the plasma membrane is activated and primed for autophosphorylation (40). However, none of these previous studies demonstrated the ability of the integrin β1 cytoplasmic tail to restore a cell’s ability to form tumors independently of its extracellular domain.

Adhesion-independent integrin signaling can promote tumor survival and growth. For example, the extracellular domain of integrin α3β1, via its interactions with CD151 but independent of binding to laminin-332, can provide essential survival signals that control skin carcinogenesis (41). In addition, in tumor xenografts, unligated integrin αvβ3 interacts with galectin-3 at the plasma membrane, resulting in recruitment of KRAS and RalB. This ECM-independent clustering leads to the downstream activation of TBK1 and NF-κB, which regulates tumor initiation and anchorage-independent growth (42). In these examples, the extracellular domains of the integrins interact with CD151 or galectin-3, thus promoting integrin signaling independent of binding to ECM. In contrast, our data suggest a mechanism of integrin β1 cytoplasmic tail signaling that can be propagated independently of the extracellular domain. Work by others has demonstrated that increased integrin expression in suprabasal skin epithelial cells not in contact with the basement membrane (and presumably not in contact with other ECM components) can lead to increased tumor formation in a mouse skin carcinogenesis model (43). However, these tumors arise from basal cells, suggesting that the mechanism is altered communication between the suprabasal and basal skin cells, possibly via a TGF-β–dependent mechanism (43). Thus, this is a distinct mechanism from that proposed in this manuscript.

We found that cells containing Y-to-A mutations at residues Y783 and Y795 in the cytoplasmic tail of integrin β1 (KO.YYAA) produced no colonies in soft agar, produced significantly less tumor burden in mice, and failed to restore FAK phosphorylation and gene expression patterns seen in the WT cells. This is consistent with studies where these Y-to-A mutations rendered phenotypes that were similar to an integrin β1–null phenotype in constitutive knock-in models (44), as well as in tissue-specific knock-in models targeting the skin (44) and collecting system of the kidney (4). The tyrosines in these motifs are important in facilitating induced-fit protein-to-protein interactions of multifunctional integrin-binding proteins like kindlins that are required for integrin activation and signaling (4). It is likely that the KO.YYAA mutants cannot activate FAK as kindlin-2 is necessary for FAK activation via formation of a kindlin/paxillin/FAK complex (45). Understanding these protein complexes that facilitate integrin β1 cytoplasmic tail–dependent signaling is critical for the rational design of new integrin-targeted therapeutics.

We found that increased integrin β1 expression in human lung adenocarcinoma tumors is significantly associated with increased tumor size, which supports our data that integrin β1 provides signaling that promotes tumor growth. In addition, the lung adenocarcinoma TCGA cohort, where increased integrin β1 expression correlated with cancer associated gene expression pathways, supports the association between integrin β1 expression and aggressive cancer in humans. These observations are consistent with other studies where integrin β1 overexpression was shown to be an independent prognostic factor for lung adenocarcinoma, and its expression correlates with an aggressive lung adenocarcinoma phenotype (8, 23, 46, 47).

Classically integrins are thought to be activated by intracellular signaling, after which they bind to a multivalent ECM ligand leading to integrin clustering and focal adhesion formation (48). The focal adhesions form a hub that informs a cell about the physical and biochemical nature of its surroundings, facilitates cell adhesion, and enables numerous well described integrin-dependent cell functions. In addition, integrin-dependent focal adhesion formation is required for maximum activation of growth factor receptors and consequent cell proliferation (49). Integrin β1 mutants that promote tumor formation have been shown to increase integrin affinity for ECM components, leading to increased, nonspecific ligand binding that would presumably mimic ligand-dependent integrin signaling (50, 51). These classic integrin functions fail to explain why the integrin β1 cytoplasmic tail can restore the malignant phenotype in cells lacking endogenous integrin β1 and suggest that oncogenic drivers such as activated KRAS facilitate integrin-dependent signaling independent of cell adhesion (48). This is consistent with previous data where activated RRAS was shown to activate integrins and promote integrin-mediated cell adhesion (52). Thus, in the setting of cancer, it is possible that constitutively active Ras proteins facilitate nonadherent cells to form a signaling hub around the integrin cytoplasmic tail by a mechanism that does not require integrin clustering. While this hypothesis requires further testing, it is clear that the integrin cytoplasmic tail functions as a key node integrating signaling that is critical to the transformed phenotype (48).

In conclusion, our data suggest that, just as nontransformed epithelial cells require integrin-mediated adhesion signaling for survival, *KRAS-*mutated lung adenocarcinomas maintain this requirement for cell survival and proliferation. We further show in cancer cells that this signaling can be provided independent of cell adhesion or integrin β1–ECM ligation via the integrin β1 cytoplasmic tail, thus facilitating the malignant phenotype independently of integrin-ECM ligation. These findings suggest that antiintegrin cancer therapies need to target the cytoplasmic tail to be successful.

## Methods

Supplemental Methods are available online with this article.

### Urethane transgenic mouse model.

We crossed integrin β1^fl/fl^ mice on an FVB background with universal deleter Vasa-Cre mice to generate integrin β1^fl/0^ mice. We then crossed integrin β1^fl/0^ mice with mice with dox-inducible Cre recombinase under control of the SPC promoter (18). Dox chow (200 mg/kg) was introduced at 4 weeks of age (Bio-Serv, S3888). Tumorigenesis was initiated with i.p. urethane (ethyl carbamate, MilliporeSigma, U2500, 1.0 mg/kg) at 8 weeks. Mice were sacrificed at approximately 42 weeks or per humane endpoints. See Supplemental Methods for further detail, including those regarding histology, tissue staining, and the tamoxifen mouse model. All mice were obtained from the Jackson Laboratory.

### Single-cell RNA-Seq.

Tumors and normal adjacent tissue of 2 WT and 2 integrin β1–KO mice were macrodissected and dissociated using the Miltenyi Biotec gentleMacs dissociator and the mouse tumor dissociation kit (Miltenyi Biotec, 130-096-730). In total, 10,000 viable cells were captured for each tissue. Single-cell RNA-Seq libraries were prepared using the 10X Chromium Single Cell Platform (10X Genomics, catalogs 1000006, 1000080, and 1000020) following the manufacturer’s protocol. The libraries were sequenced using the NovaSeq 6000 with 150 bp paired end reads. RTA (version 2.4.11; Illumina) was used for base calling, and analysis was completed using 10X Genomics Cell Ranger software v2.1.1. The FASTQ and matrix files have been uploaded to NCBI Gene Expression Omnibus (https://www.ncbi.nlm.nih.gov/sra), series GSE175687. See Supplemental Methods for details regarding single-cell RNA-Seq analysis.

### Xenograft mouse model.

Eight-week-old athymic mice (*Foxn1^nu^*) were purchased from the Jackson Laboratory (stock no. 002019-Nu/J). In total, 1 × 10^6^ cells were suspended in Matrigel (1 mg/mL) and injected into the left lung. At 45 days, mice were euthanized and heart/lungs resected en bloc. Lungs were paraffin embedded, sectioned every 100 μm, and H&E stained. Images were obtained, and tumor area per high-power field was measured using ImageJ software (version 1.52; NIH). For bioluminescence experiments, cells were labeled with luciferase^+^ lentivirus (System Biosciences, BLIV713VA-1), mice were administered 30 mg/mL luciferin (Perkin-Elmer, 122799), and bioluminescence was measured using the Perkin-Elmer IVIS Spectrum bioluminescent and fluorescent imaging system prior to euthanasia.

### Statistics.

Statistical analyses, unless stated otherwise, were performed with GraphPad Prism version 9.0.0. An unpaired, 2-tailed *t* test was used single comparisons and Sidak’s multiple-comparison test for multiple comparisons. *P* < 0.05 was considered significant. Data are shown as mean ± SEM.

### Study approval.

All animal experiments were approved by the Vanderbilt University Medical Center IACUC. Mice were housed in an AAALAC-accredited facility with a standard 12-hour light/dark schedule and fed regular chow diet, unless stated otherwise. Human studies were approved by the Vanderbilt IRB, and written informed consent was received from living participants prior to inclusion in the study.

## Author contributions

SMH contributed by designing research studies, conducting experiments, acquiring data, analyzing data, providing reagents, and writing the manuscript; EJP contributed by designing research studies, conducting experiments, and providing reagents; JAK contributed by designing research studies, conducting experiments, analyzing data, and providing reagents; LAV contributed by conducting experiments, acquiring data, and analyzing data; AR contributed by analyzing data; FB contributed by designing research studies, conducting experiments, acquiring data, and analyzing data; BTC contributed by conducting experiments and acquiring data; AJL contributed by conducting experiments and acquiring data; KN contributed by conducting experiments and acquiring data; ZQX contributed by conducting experiments and acquiring data; RAP contributed by conducting experiments and acquiring data; SL contributed by conducting experiments and acquiring data; HT contributed by analyzing data; VVP contributed by acquiring data and analyzing data; OMV contributed by training, conducting experiments, and acquiring data; AJ contributed by designing research studies, conducting experiments, and acquiring data; WL contributed by designing research studies, conducting experiments, acquiring data, and providing reagents; MHW contributed by designing research studies and providing reagents; WKR contributed by designing research studies; PPM contributed by designing research studies; AP contributed by designing research studies; TSB contributed by designing research studies; and RZ contributed by supervision, designing research studies, providing reagents, and writing the manuscript.

## Supplementary Material

Supplemental data

## Version 1 06/28/2022

In-Press Preview

## Version 2 08/08/2022

Electronic publication
