# Supplementary material for: Ligand-independent integrin β1 signaling supports lung adenocarcinoma development
Source: JCI Insight. 2022 Aug 8;7(15):e154098. doi: 10.1172/jci.insight.154098 (PMC9462485; doi:10.1172/jci.insight.154098)
Supplement: Supplemental data [file jciinsight-7-154098-s114.pdf]

Supplementary Figures:

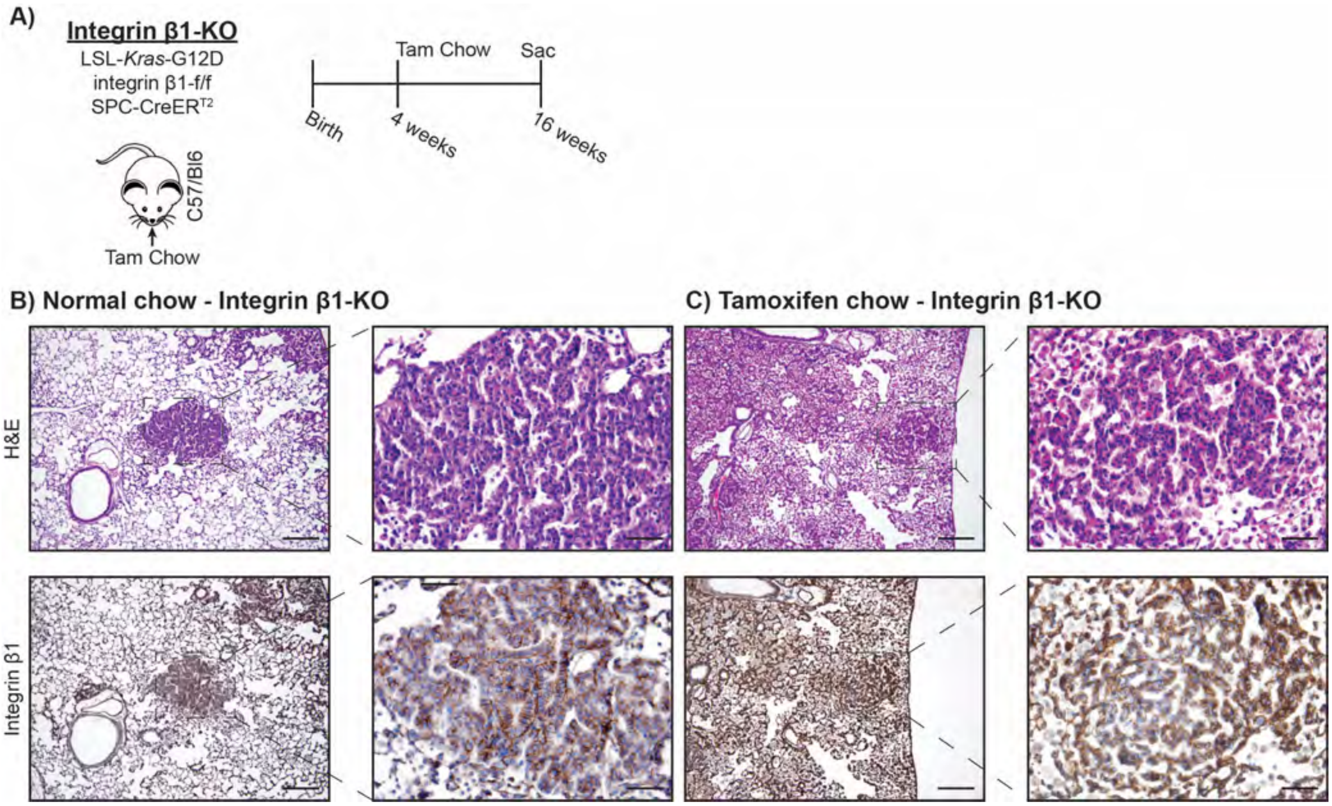

Supp. Figure 1: LSL-Kras-G12D tamoxifen model. A) LSL-*Kras*-G12D; integrin β1 $^{f/f}$; SPC-CreER^{T2} were bred and the model was set up as depicted in the diagram. **B)** Mice died prematurely due to tumor formation in the lungs of tamoxifen-naïve mice. These tumors were integrin β1 positive (lower panel). **C)** Tumor formation was increased even further in tamoxifen-exposed mice, which also stained positive for integrin β1 (lower panel). These data suggested this Cre was extremely leaky, so no further experiments were not attempted with this model.

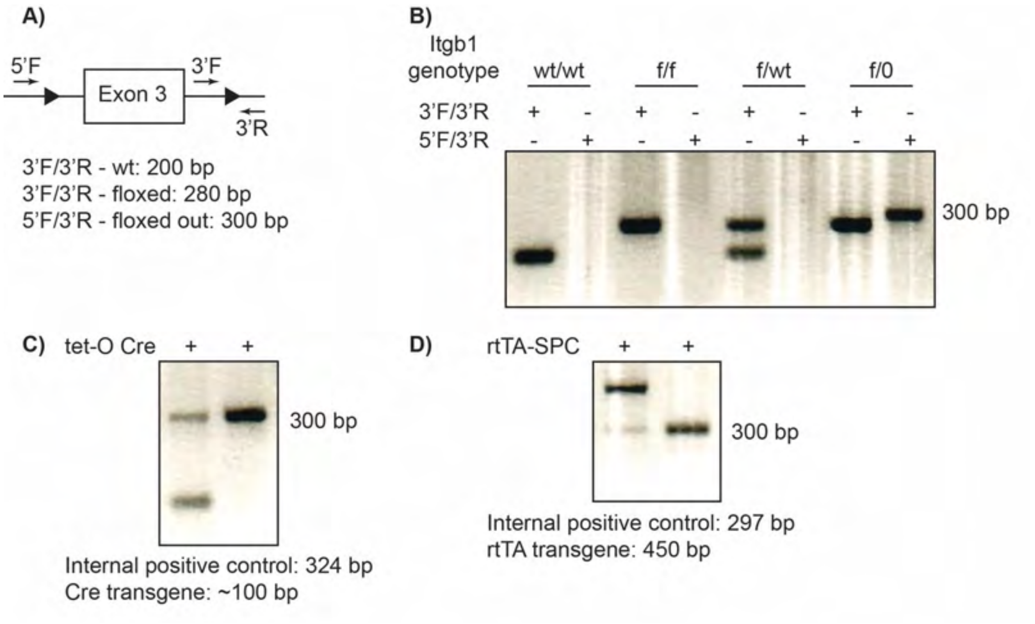

Supp. Figure 2: Genotyping results for SPC rtTA; TetO-Cre; integrin $\beta1^{f/0}$ mouse. A) Primer schema for Itgb1 alleles. **B)** Shown are four distinct genotype combinations for the Itgb1 allele. **C)** The left lane represents presence of the tet-O Cre allele, and the right lane represents its absence. **D)** The left lane represents presence of the rtTA-SPC allele, and the right lane represents its absence. The integrin $\beta1^{f/0}$ mice were selected for "WT" or "control" mice. The SPC rtTA; TetO-Cre; integrin $\beta1^{f/0}$ mice were selected for the integrin β1-KO or "KO" mice. Black lines denote margin of cropped image. "+" = presence of primers. "-" = absence of primers. bp = base pair.

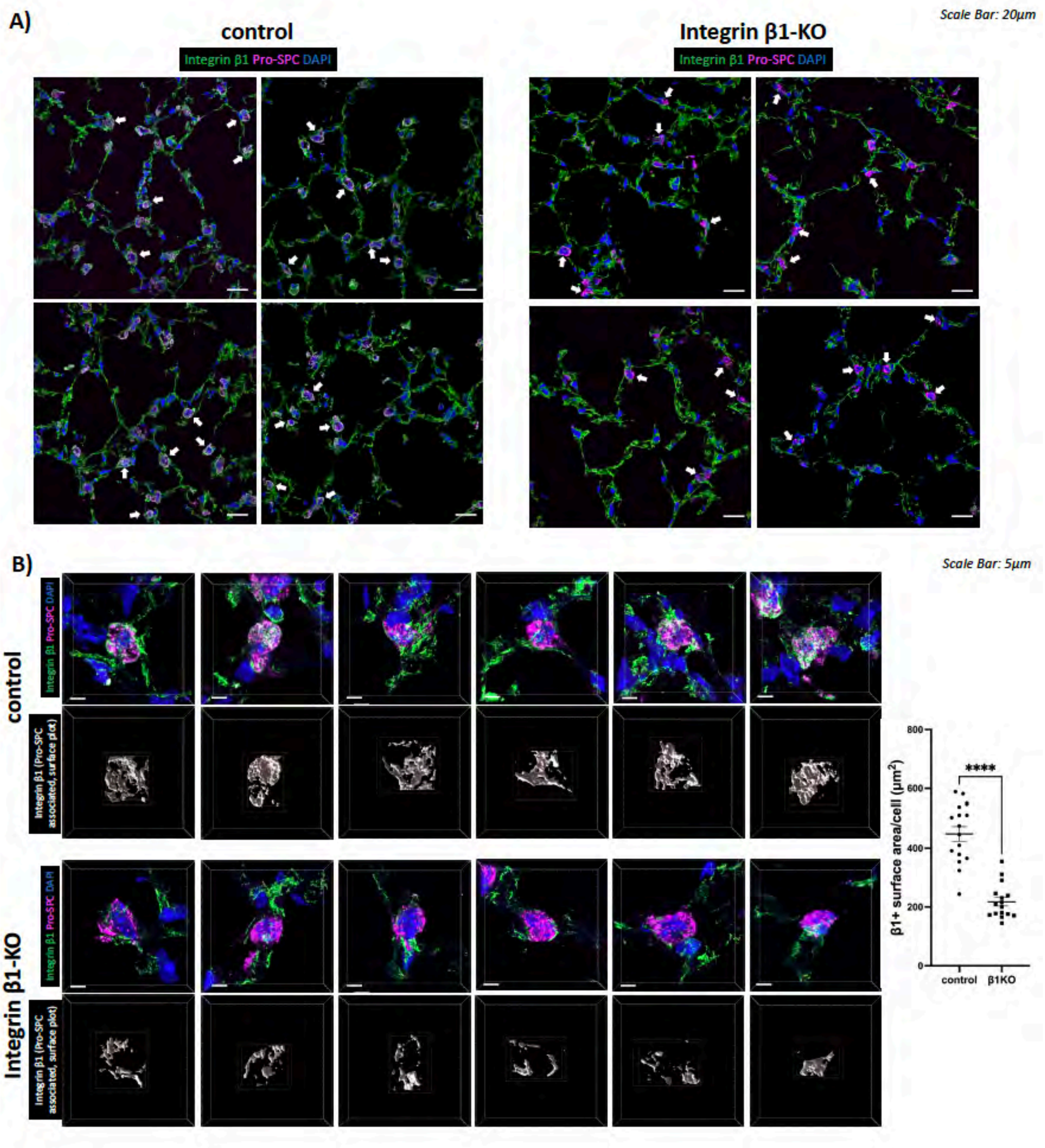

Supp. Figure 3: Integrin β1 is deleted in type 2 alveolar epithelial (AT2) cells in mice. A) Immunostaining for pro-SPC (magenta) and integrin β1 (green) in integrin β1$^{f/0}$ mice without (control) and with SPC rtTA;TetO-Cre (integrin β1-KO). Scale bar = 20 μm. White arrows mark examples of SPC-positive cells. **B)** Three-

dimensional super-resolution microscopy was performed on SPC-positive cells of control and integrin β1-KO lungs (upper panels). These images were used for three dimensional reconstructions and surface plots for integrin β1 associated with pro-SPC (lower panels). Quantification of integrin β1-positive surface areas was compared. Scale bar = 5 μm. Graph shows mean +/- SEM, n=3 mice for each genotype, only 3D conformations graphed. ****$p<0.0001$ by unpaired, two-tailed *t* test.

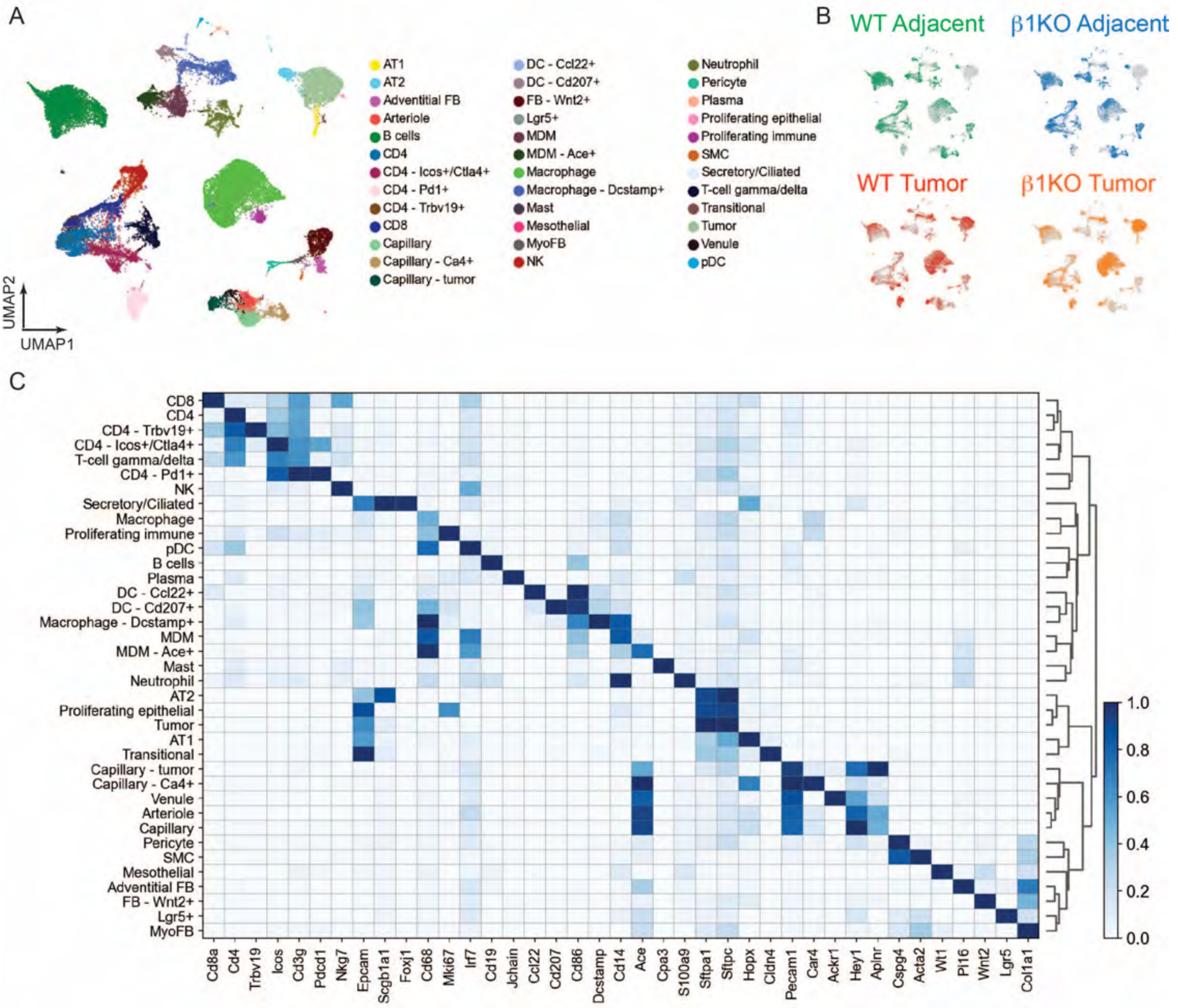

Supp. Figure 4: Cell types and discriminating marker expression identified in single cell RNA-seq experiment. A) Uniform manifold approximation and projection (UMAP) depicting 41,494 cells isolated from tumors or adjacent tissue from integrin β1-KO and control mice after urethane. **B)** Corresponding cell types are shown in relative proportion in control and integrin β1-KO mice for both tumor and adjacent normal tissue. **C)** Heatmap demonstrating key discriminating marker expression across cell types.

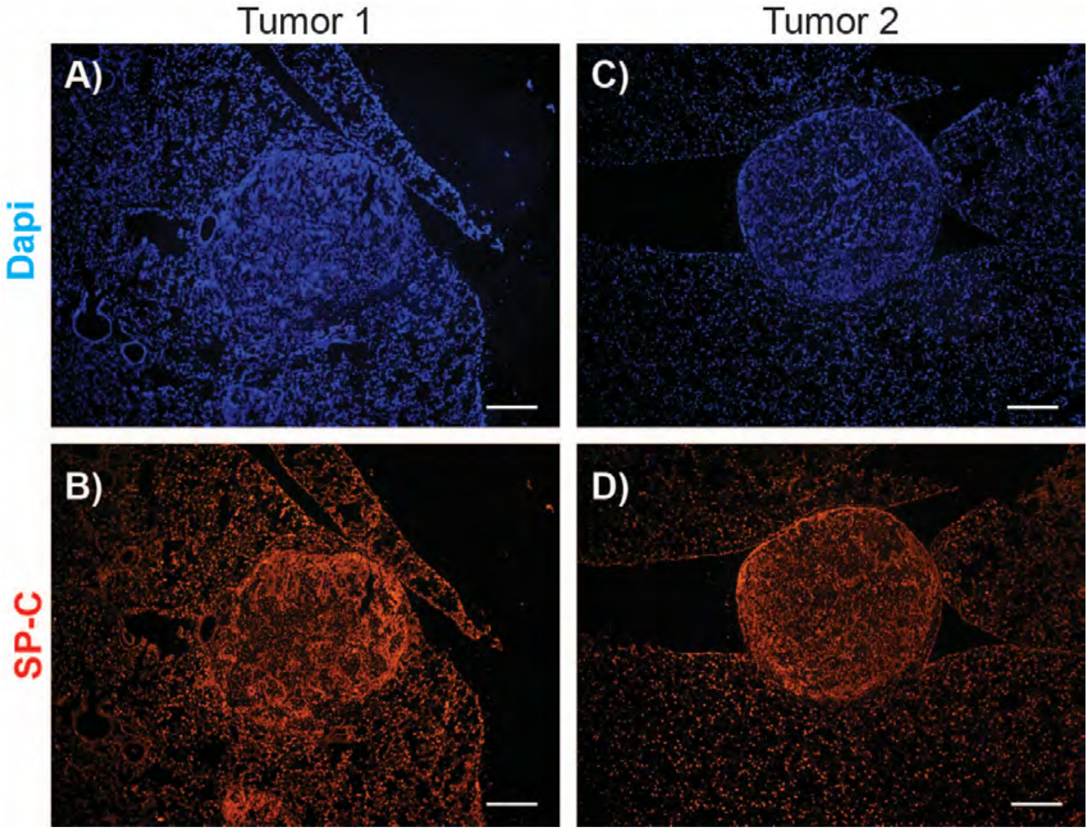

Supp. Figure 5: Urethane-induced tumors in integrin β1-KO mice express pro-SPC. Frozen sections from urethane-induced tumors that developed in SPC rtTA; TetO-Cre; integrin $\beta1^{f/0}$ mice treated with doxycyline (i.e., integrin β1-KO mice) were stained with **A & C)** DAPI and for **B & D)** SPC. Tumors express pro-SPC, which is consistent with the cells of origin being SPC-positive type 2 alveolar epithelial cells.

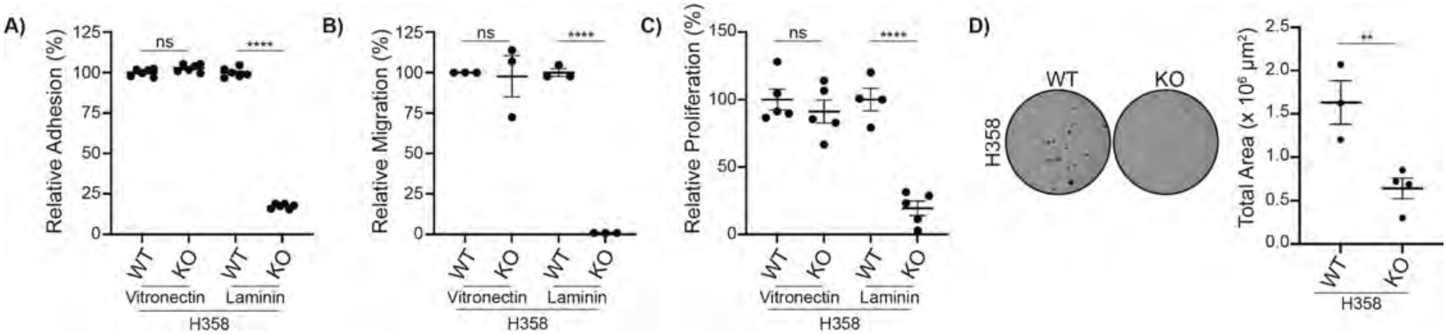

Supp. Figure 6: Deletion of integrin β1 in H358 human lung cancer cells results in decreased adhesion, migration, proliferation and colony formation. The WT and integrin β1-KO H358 cells were plated on integrin β1-independent (vitronectin) and -dependent (laminin I) matrices. Relative **A)** adhesion, **B)** migration, **C)** BRDU proliferation and **D)** colony formation (representative photomicrographs and quantification) are graphed for WT and integrin β1-KO cells (n=3 replicates). *p<0.05; **p<0.01; ***p<0.001; ****p<0.0001; ns = p>0.05. Graphs show mean +/- SEM.

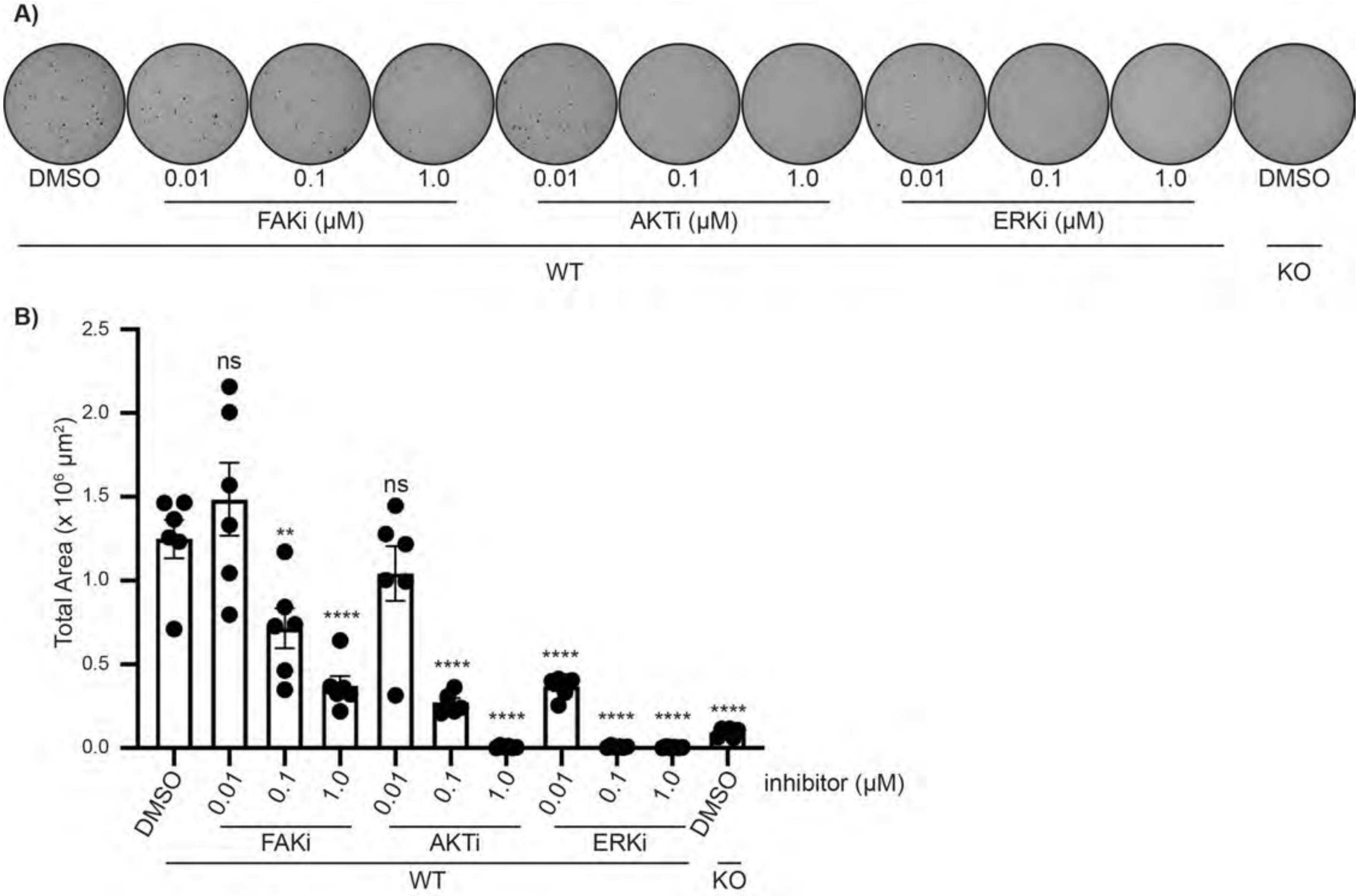

Supp. Figure 7: Inhibition of FAK, AKT and ERK in A549 human lung adenocarcinoma cells reduces colony formation in soft agar assay. WT A549 cells were treated with inhibitors of FAK (defactinib), AKT (MK-2206) and ERK (SCH772984) at the specified doses. Representative **A)** photomicrographs and **B)** surface area quantification of colonies are shown. *p<0.05; **p<0.01; ***p<0.001; ****p<0.0001; ns = p>0.05 by by Sidak's multiple comparison test. Graphs show mean +/- SEM. FAKi = FAK inhibitor. AKTi = AKT inhibitor. ERKi = ERK inhibitor.

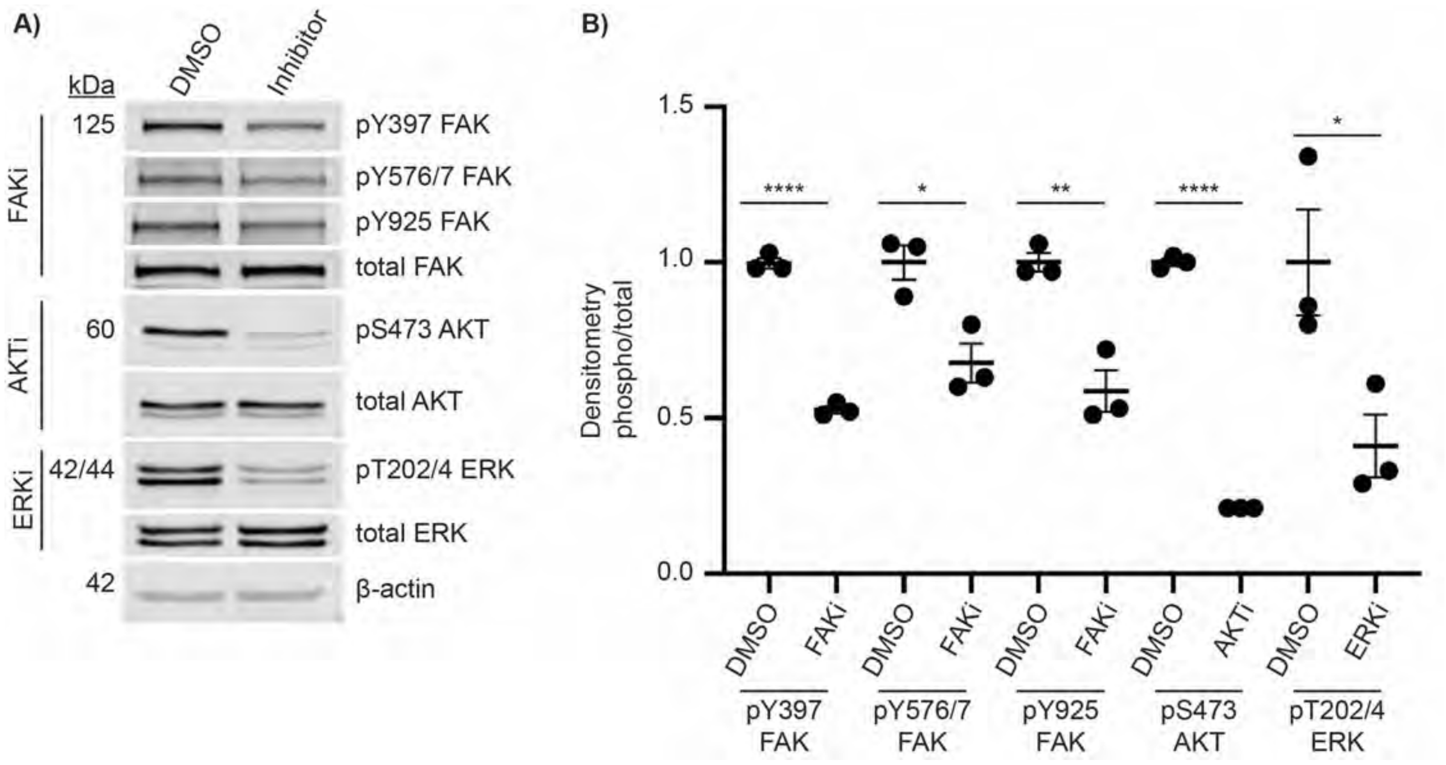

Supp. Figure 8: Inhibitors to FAK, AKT and ERK decrease kinase phosphorylation. A549 cells were treated with inhibitors of FAK (defactinib 1.0 μM), AKT (MK-2206 0.1 μM) and ERK (SCH772984 0.01 μM) for 1 hour. **A)** Phosphorylation of the kinases was measured by Western blot. **B)** The ratio of densitometry values for the phosphorylated and total protein are plotted (n=3 replicates). *p<0.05; **p<0.01; ***p<0.001; ****p<0.0001; ns = p>0.05 by unpaired, two-tailed *t* test. Graphs show mean +/- SEM. FAKi = FAK inhibitor. AKTi = AKT inhibitor. ERKi = ERK inhibitor.

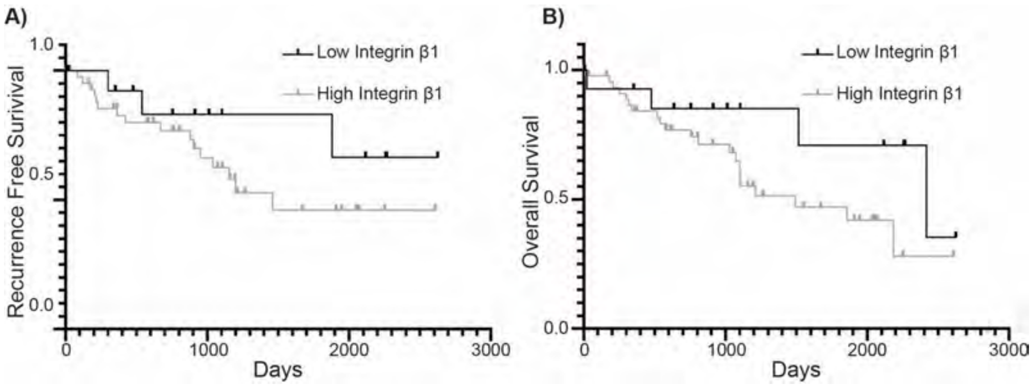

Supp. Figure 9: Tumors with high integrin β1 protein expression trend towards inferior recurrence free survival and overall survival. A) Recurrence free and **B)** overall survival was compared in patients with high (score 2-3 staining intensity) versus low integrin β1 protein expression (score 0-1 staining intensity) using a Kaplan-Meier curve.

Demographic Characteristics	
N (Total)=	71
AGE at Block Collection (SD)	66.5(8.5)
Gender	
M	22(31%)
F	49(69%)
Race	
White	69(97%)
Black	2(3%)
BMI (SD)	26.9(5.8)
Smoking status	
Current	13(18%)
Former	47(66%)
Never	11(16%)
Age Started (SD)	18.8(6.5)
Age Quit (SD)	50.1(15.4)
Pack years of smoking (SD)	44.7(29.8)
Asbestos Exposure	
Yes	5(7%)
No	66(93%)
Prior Cancer	
Yes	39(55%)
No	32(45%)
Nodule Size (mm) (SD)	25.9(14.6)
Nodule Location	
LUL	16(23%)
LLL	12(17%)
RUL	19(27%)
RML	4(6%)
RLL	20(28%)
PET avidity (suv)	
Positive	49(69%)
Negative	16(23%)
N/A	4(6%)
Not Available	2(3%)
Diagnosis Age, years (SD)	66.5(8.5)
Performance Status at dx (ECOG)	
Grade 0	61(86%)
Grade 1	7(10%)
Not Available	3(4%)
Path Stage	
Stage IA	28(40%)
Stage IB	18(25%)
Stage IIA	7(10%)
Stage IIB	6(8%)
Stage IIIA	11(16%)
Stage IIIB	1(1%)
Primary Treatment	
Surgery	71(100%)
FEV1% (AVG ± STDV)	(86.8 ± 18.8)

Supp. Table 1: Tissue microarray characteristics. The lung adenocarcinoma tissue microarray was designed with 71 patients, though no tumor was available for analysis for 6 patients. All patient tissue was procured at Vanderbilt University Medical Center.

Supplementary methods:

Urethane transgenic mouse model. We crossed integrin $\beta 1^{f/f}$ mice on an FVB background with universal deleter Vasa-Cre mice to generate integrin $\beta 1^{f/0}$ mice. We then crossed integrin $\beta 1^{f/0}$ mice with mice with inducible Cre recombinase expression by the dox-inducible reverse tetracycline activator under control of the SPC promoter (18). AT2 deletion was introduced at four weeks of age using dox chow 200 mg/kg (Bio-Serv #S3888). Control $\beta 1^{fl/0}$ mice were also fed dox chow. Tumorigenesis was initiated with intraperitoneal urethane (ethyl carbamate, Sigma #U2500, 1.0 mg/kg) at eight weeks. Mice were sacrificed at approximately 42 weeks or upon reaching humane endpoints. All mice were obtained from Jax.com.

LSL-Kras-G12D; integrin $\beta 1^{f/f}$; SPC-CreER^{T2} tamoxifen inducible mouse model. We crossed the inducible LSL-Kras-G12D allele and the tamoxifen-inducible Cre recombinase allele onto integrin $\beta 1^{f/f}$ mice. All mice were C57/Bl6 background and obtained from Jax.com. Tamoxifen chow 400 mg/kg (Envigo #TD.130860) was started at four weeks of age (5 days tamoxifen chow, 2 days normal chow cycles). Mice were sacrificed approximately 16 weeks after tamoxifen chow or upon reaching humane endpoints.

Histology and tissue staining. Lungs were inflation fixed at 25 cm with 10% formalin, sectioned, and H&E stained. Integrin β1 immunohistochemistry of mouse tumors was performed on paraffin sections incubated with primary antibody (rabbit anti-integrin β1, Cell Signaling Technology (CST), Danvers, MA, USA #34971) and anti-rabbit secondary and DAB (#8114). For frozen sections, lungs were inflation fixed with a 2:1 mixture of PBS:OCT, embedded in OCT and flash frozen. Integrin β1 and pro-SPC were stained with primary antibodies (rat anti-integrin β1, Millipore, Burlington, MA, USA #1997; rabbit anti-pro-SPC, Abcam, Cambridge, UK #90716) and secondary antibodies (donkey anti-rat, Alexa 488, Life Technologies, Carlsbad, CA, USA #21208; donkey anti-rabbit, Alexa 555, Life Technologies #A31572) per manufacturer protocols. Images were obtained using a Nikon Spinning Disk TiE inverted fluorescence confocal microscope attached to an Andor DU-897 EMCCD camera (x60 objective). Three-dimensional super-resolution microscopy was performed using a Zeiss LSM 980 confocal microscope with an Airyscan 2 detector and a 63x/1.40 Plan-Apochromat (Oil) objective. Stacks were acquired with 50-60 images per stack and ~1 image/0.15µm. Post-imaging processing (spectral unmixing) was performed using ImageJ/Fiji. 3D reconstructions and surface plots were created using Imaris Software.

Single-cell RNA-seq. Tumors and normal adjacent tissue of two WT and two integrin β1-KO mice were macrodissected and dissociated using the Miltenyi Biotec gentleMacs dissociator and the mouse tumor dissociation kit (Miltenyi Biotec #130-096-730). Tissue pellets were strained through 100 μm then 70 μm filters (Stemcell Technologies #27217 and 27216) and RBC lysis performed per manufacturer's protocol (RBC lysis buffer, Gibco #A10492-01ACK). Cells were stained with propidium iodide (Sigma #P4864). Approximately 10,000 viable cells were captured for each tissue. scRNA-seq libraries were prepared using the 10X Chromium Single Cell Platform (10X Genomics #1000006, 1000080, and 1000020) following the manufacturer's protocol. The libraries were sequenced using the NovaSeq 6000 with 150 base pair paired end reads. RTA (version 2.4.11; Illumina) was used for base calling and analysis was completed using 10X Genomics Cell Ranger software v2.1.1. The FASTQ and matrix files have been uploaded to NCBI Gene Expression Omnibus (https://www.ncbi.nlm.nih.gov/sra), series GSE175687.

Single-cell RNA-seq analysis. After alignment and demultiplexing, samples were jointly analyzed using a standard Seurat (54)/Scanpy (55) pipeline as previously described (56). Briefly, cells containing fewer than 500 genes, <0.5% or >10% mitochondrial reads were filtered and excluded from downstream analysis. Libraries were merged and jointly normalized and scaled using SCTransform (56) in Seurat v3.2 including "percent.mt" as a regression variable, followed by principal components analysis using variable genes and graph-based clustering. Immune (*Ptprc*+), epithelial (*Epcam*+) and stromal (*Pecam1*+ or *Col1a1*+) cells were independently extracted, followed by recursive clustering, doublet exclusion (clusters containing nonphysiologic marker combinations), and cell-type annotation. Immune, epithelial and stromal objects were then merged, uniform manifold approximation and projection (UMAP)-embedded (57). Differential expression analysis was performed using the FindMarkers tool in Seurat using the Wilcoxon test. Visualization and presentation were performed using Scanpy v.1.51. Code used for these analyses is available at www.github.com/kropskilab/itgb1_tumor/.

Cell lines. A549 and H358 cell lines were a gift from Dr. Christine Lovly (Vanderbilt-Ingram Cancer Center, Nashville, TN, USA) and are available for ATCC. Cell lines were cultured in RPMI1640 (Gibco, #11875-093) with

10% FBS plus 1% antibiotic, routinely tested for mycoplasma contamination, and authenticated with short-tandem repeat analysis (Genetica, Burlington, NC, USA).

CRISPR. ITGB1 (integrin β1) was KO of the A549 and H358 cells using CRISPR/Cas9. We followed published protocols to engineer the CRISPR/Cas9 plasmids (58). Briefly, guide RNAs were designed to exon 2 of human integrin β1 gene. These included the guide RNA used in the integrin β1-KO (guide RNA set #1, top = 5'-CACCGTTACAACCAATTTTCTGGAT-3' and bottom = 5'- AAACATCCAGAAAATTGGTTGTAAC-3') and integrin β1-KO.1 cells (guide RNA set #2, top = 5'- CACCGTGAATTTACAACCAATTTTC-3' and bottom = 5'-AAACGAAAATTGGTTGTAAATTCAC-3'). These guide RNAs were cloned into the PX-458 vector purchased from www.addgene.com (58). Cells were transfected using Lipofectamine 2000 (Thermo #11668). Cells were stained with anti-integrin β1 primary antibody (rat anti-human AIIB2 clone, University of Iowa, Iowa City, Iowa, USA) and secondary antibody (Invitrogen, Waltham, MA, USA #A10545). Cells were sorted via fluorescence-activated cell sorting (FACS) and cells that stained negative for integrin β1 (henceforth referred to as integrin β1-KO) were collected.

Western blotting. Western blotting was performed as described in our previous manuscripts (5, 59). Briefly, protein was extracted from cells, electrophoresed in a 10% SDS-PAGE gel and transferred onto nitrocellulose membranes. Membranes were blocked and incubated with primary antibody (anti-integrin β1, Millipore #AB1952; anti-pY397 FAK, CST #3283; pY576/7, FAK CST #3281; anti-pY925, CST #3284; total FAK, CST #3285; anti-pS473 AKT, CST #9271; total AKT, CST #9272; anti-pT202/4 ERK, CST #9101; total ERK, CST #9102; beta-actin, CST #3700). Membranes were incubated with secondary antibodies (goat anti-rabbit 800CW, Licor, Lincoln, NE, USA #926-32211 or donkey anti-mouse 680LT, Licor #926-68022). Signal was detected using a LI-COR Odyssey CLx Near-Infrared Western Blot Detection system.

Cell adhesion, migration, and proliferation assays. Cell adhesion and migration assays were performed as described previously (59). The migration assay used transwells with 8.0 μm pores (Costar #3422). BRDU-incorporation cell proliferation assays were used according to manufacturer protocols (Exalpha #X1327K2). These assays used plates and/or inserts coated with Matrigel (Corning, Corning, NY, USA #356230, 10 μg/mL),

laminin I derived from Engelbreth-Holm-Swarm sarcoma (20 μg/mL, Invitrogen #23017-015), or vitronectin (0.5 μg/mL, Advanced Biomatrix, Carlsbad, CA, USA #5051).

Soft agar assays. 1,500 cells were suspended in 0.5 mL of 0.35% soft agar in RPMI (Difco, Waltham, MA, USA #214220) and plated in a 24 well plate on top of a base layer of 0.5 mL of 0.5% soft agar. Media was changed 3X/week and on day 21 the cells were stained with 0.5 mL of 2.5 mg/mL MTT (Sigma #M2128). The plate was imaged with GelCount colony counter (Oxford Optronix, Abingdon, OX, UK) and analyzed using GelCount software version 1.2.1.0. The FAK, AKT and ERK inhibitors were purchased from SelleckChem (Houston, TX, USA; FAK inhibitor VS-6063, #S7654; AKT inhibitor MK-2206, #S1078; ERK inhibitor SCH772984, #S7101).

Xenograft mouse model. Eight-week-old athymic mice (*Foxn1^{nu}*) were purchased from Jackson Laboratory (#002019-Nu/J). 1 x 10^6 cells were suspended in Matrigel (Corning, Corning, NY, USA #356230, 1 mg/mL) and injected into the left lung. At 45 days, mice were euthanized and heart/lungs resected en-bloc. Lungs were paraffin embedded, sectioned every 100 μm, and H&E stained. Images were obtained and tumor area per high power field was measured using ImageJ software (version 1.52). For bioluminescence experiments, cells were labelled with luciferase-positive lentivirus (System Biosciences, # BLIV713VA-1), mice were administered 30 mg/mL luciferin (Perkin-Elmer #122799) and bioluminescence measured using the Perkin-Elmer IVIS Spectrum bioluminescent and fluorescent imaging system prior to euthanasia.

Bulk RNA-seq and data analysis. RNASeq libraries were prepared using 300 ng of RNA and the NEBNext® Ultra™ II RNA Library Prep kit (NEB #E7760L) per manufacturer's instructions, with mRNA enriched via poly-A-selection using oligoDT beads. The RNA was then thermally fragmented and converted to cDNA, adenylated for adaptor ligation and PCR amplifed. Individual libraries were assessed for quality using the Agilent 2100 Bioanalyzer and quantified with a Qubit Fluorometer. The adapter ligated material was evaluated using qPCR prior to normalization and pooling for sequencing.

RNASeq libraries were prepared using 300 ng of RNA and the NEBNext® Ultra™ II RNA Library Prep kit (NEB, Ipswich, MA, USA #E7760L) per manufacturer's instructions. The libraries were sequenced using the NovaSeq

6000 with 150 base pair paired end reads. The FASTQ files have been uploaded to NCBI Sequence Read Archive (https://www.ncbi.nlm.nih.gov/sra), BioProjectID SUB9677957.

The libraries were sequenced using the NovaSeq 6000 with 150 base pair paired end reads. RTA (version 2.4.11; Illumina) was used for base calling and data QC was completed using MultiQC v1.7 by the Vanderbilt Technologies for Advanced Genomics (VANTAGE) core (Vanderbilt University, Nashville, TN). The FASTQ files have been uploaded to NCBI Sequence Read Archive (https://www.ncbi.nlm.nih.gov/sra), BioProjectID SUB9677957.

RNA-seq analysis. Dragen pipeline was run on Basespace to perform QC and analyze the RNA-seq fastq files to generate read counts for each of the samples (60). Genes with very low counts (sum across samples <=10) were excluded. DESeq2 was used to perform two sets of differential gene expression analysis: 1) comparing integrin β1-KO vs. WT, and 2) identifying genes associated with the rescue phenotype: by comparing KO.ITGB1, KO.Tacβ1 and KO.YYAA versus integrin β1-KO. Pathway enrichment analysis was performed using KEGG pathways from MSigDB (msigdbr package (61)) using Fisher's exact test. Heatmaps were plotted using ComplexHeatmap package (62). These analyses were performed using R version 4.0.3.

Integrin β1 expression in integrin β1-KO cells. The KO.ITGB1, KO.Tacβ1 and KO.YYAA A549 cells were engineered by re-expressing full integrin β1 or its various constructs in the integrin β1-KO cells. Full-length integrin β1 was expressed in the integrin β1-KO cells to create the KO.ITGB1 cells. The Tacβ1 chimeric gene was expressed in the integrin β1-KO cells to create the KO.Tacβ1 cells. Integrin β1 containing Y-to-A cytoplasmic tail mutations at residues Y783 and Y795 was expressed in integrin β1-KO cells to create the KO.YYAA cells. These genes were cloned using standard molecular biology techniques into the PB-CMV-MCS-EF1α-GreenPuro *piggyBac* transposon vector (SBI #PB513B-1). All vector sequences were confirmed with DNA sequencing. Cells were transfected with both the expression vector and pCMV-m7pB transpose vector (63). Cells were collected via flow cytometry that expressed either the integrin β1 ectodomain (KO.ITGB1 and KO.YYAA) or the Tac domain (KO.Tacβ1). A mouse anti-human IL-2R PE-conjugated antibody was used to stain the KO.Tacβ1 cells (R&D Systems #FAB1020P).

Patient data and tissue microarray staining. The tissue microarray is composed of tumors spotted in duplicate from 65 deidentified patients from Vanderbilt University Medical Center (Nashville, TN, USA). Slides were placed on the Leica Bond Max IHC stainer. All steps besides dehydration, clearing and cover slipping were performed on the Bond Max. Slides are deparaffinized. Heat induced antigen retrieval was performed on the Bond Max using their Epitope Retrieval 1 solution for 20 minutes. Slides were incubated with anti-integrin β1 (CST #34971) or isotype control. The Bond Polymer Refine system was used for visualization. Slides were the dehydrated, cleared and cover slipped.

TCGA data analysis. TCGA lung adenocarcinoma (LUAD) data was analyzed for *ITGB1* (integrin β1) gene expression and its association with the expression of other genes/pathways and survival data. TCGA LUAD data was downloaded from www.cbioportal.org. Integrin β1 gene expression data was modeled as a mixture of gaussians to identify a high versus low expression groups using the mixtools package in R. Survival analysis was performed to identify the association of the integrin β1 expression groups versus overall survival. Correlation analysis was performed to identify genes significantly correlated with integrin β1 expression (adjusted p-value < 0.001). Geneset enrichment was performed using KEGG pathways from msigdbr R library.

Statistical analyses. Statistical analyses, unless stated otherwise, were performed with GraphPad Prism version 9.0.0. Please see methods for details of statistics used in the analysis of single cell RNA-seq data, bulk RNA-seq data, and clinical data. An unpaired, two-tailed *t* test was used single comparisons and Sidak's multiple comparisons test for multiple comparisons. A P value of <0.05 was considered significant. Error bars represent standard error of the mean (SEM).

Study approval. All animal experiments were approved by the Vanderbilt University Medical Center Institutional Animal Care and Use Committee. Mice were housed in an AAALAC-accredited facility with a standard 12-hour light/dark schedule and fed regular chow diet, unless stated otherwise.
